# The proper timing of *Atoh1* expression is pivotal for hair cell subtype differentiation and the establishment of inner ear function

**DOI:** 10.1007/s00018-023-04947-w

**Published:** 2023-11-06

**Authors:** Dan You, Wenli Ni, Yikang Huang, Qin Zhou, Yanping Zhang, Tao Jiang, Yan Chen, Wenyan Li

**Affiliations:** 1 ENT Institute, Department of Otorhinolaryngology, Eye and ENT Hospital, State Key Laboratory of Medical Neurobiology, MOE Frontiers Center for Brain Science, The Institutes of Brain Science and the Collaborative Innovation Center for Brain Science, Fudan University, Shanghai, 200031 People’s Republic of China; 2 NHC Key Laboratory of Hearing Medicine, Fudan University, Shanghai, 200031 People’s Republic of China

**Keywords:** *Atoh1*, Hair cell regeneration, Maturation, Differentiation, Function

## Abstract

*Atoh1* overexpression is essential for hair cell (HC) regeneration in the sensory epithelium of mammalian auditory and vestibular organs. However, *Atoh1* overexpression alone cannot induce fully mature and functional HCs in the mammalian inner ear. In the current study, we investigated the effect of *Atoh1* constitutive overexpression in native HCs by manipulating *Atoh1* expression at different developmental stages. We demonstrated that constitutive overexpression of *Atoh1* in native vestibular HCs did not affect cell survival but did impair vestibular function by interfering with the subtype differentiation of HCs and hair bundle development. In contrast, *Atoh1* overexpression in cochlear HCs impeded their maturation, eventually leading to gradual HC loss in the cochlea and hearing dysfunction. Our study suggests that time-restricted *Atoh1* expression is essential for the differentiation and survival of HCs in the inner ear, and this is pivotal for both hearing and vestibular function re-establishment through *Atoh1* overexpression-induced HC regeneration strategies.

**Supplementary Information:**

The online version contains supplementary material available at 10.1007/s00018-023-04947-w.

## Introduction

Sensory hair cells (HCs) are delicate mechano-sensors in vertebrate vestibular and auditory organs, and these cells are vulnerable to noise, ototoxic drugs, infections, and other sources of damage. Although there is limited capacity for HCs to regenerate in the mature vestibule and the neonatal cochlea following injury in mammals [1–3], HCs cannot be regenerated in sufficient numbers to restore inner ear function, which leads to permanent balance disorders or sensorineural hearing loss [1–4]. Supporting cells (SCs) are believed to share common progenitors with HCs [5–7], and SCs are regarded as the best cellular resource for HC regeneration after injury [8, 9]. Therefore, understanding the molecular mechanisms underlying HC fate determination, maturation, and survival will benefit regeneration strategies.

*Atoh1*, which encodes a basic helix-loop-helix transcription factor, is one of the most important genes known to be necessary for the differentiation of inner ear HCs [7, 10, 11]. Embryonic *Atoh1*-null mice fail to generate vestibular and cochlear HCs [7], and conditional deletion of *Atoh1* in *Pax2*^+^ progenitor cells at the embryonic stage leads to the loss of HCs and the subsequent degeneration of the organ of Corti [12]. In the vestibular sensory epithelium, *Atoh1* is expressed from embryonic day (E)12.5 and is downregulated once HCs mature in type I HCs, but continues to be expressed in type II HCs [10, 13, 14]. During cochlear development, the *Atoh1* level peaks at E17.5, then decreases from the postnatal period in the cochlea, and becomes almost undetectable in mature auditory HCs [15, 16]. Conditional knockout of *Atoh1* at different points in HC development from E15.5 to E17.5 also leads to HC loss and defects [17–20]. However, the significance of the timely downregulation of *Atoh1* during the development of HCs is not fully understood [21]. It is well known that *Atoh1* is a potent driver of HC differentiation in the inner ear, and ectopic expression of *Atoh1* leads to the production of new HCs in the vestibule and cochlea both in vitro and in vivo [19, 22–28]. Overexpression of *Atoh1* has now become a popular method for HC regeneration in both neonatal and adult mice [29–33]. However, functional maturation of new HCs has not yet been achieved by the ectopic expression of *Atoh1* in the inner ear sensory epithelium [22, 23, 34], which might be due to insufficient factors for the direct trans-differentiation from SCs to HCs or inhibitory effects on the maturation or survival of HCs caused by the permanent expression of *Atoh1*.

**Fig. 1:**
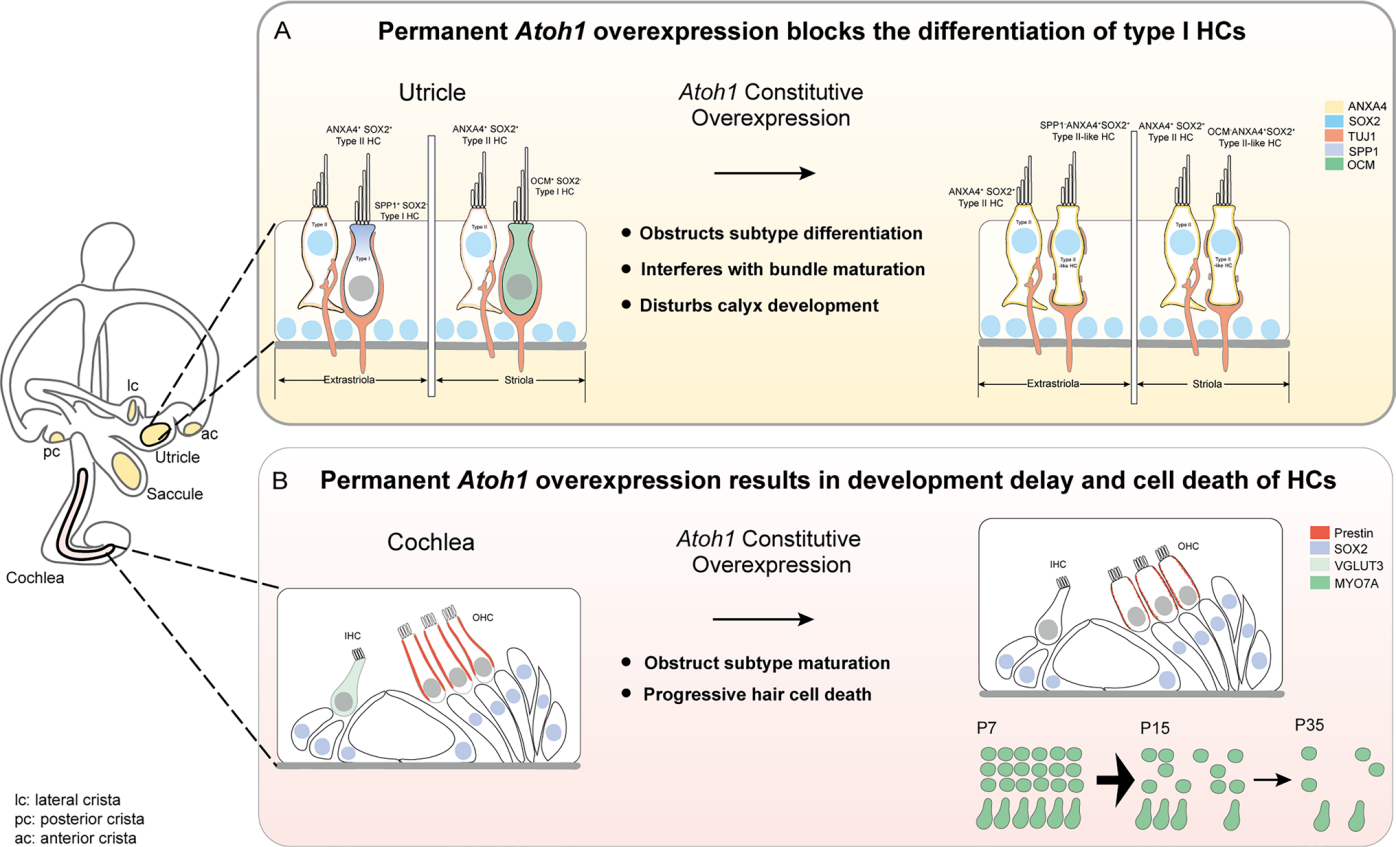
Permanent *Atoh1* overexpression blocked the differentiation of HC subtypes and led to cell death of cochlear HCs. **A** In the utricle of mature WT mice, type I HCs were flask-shaped with a narrow neck and relatively long stereocilia bundles. Type II HCs were cylindrical and had shorter bundles. Type I HCs were SPP1^+^/SOX2^−^ in the extrastriola and OCM^+^/SOX2^−^ in the striola, and with full calyx, while type II HCs were ANXA4^+^/SOX2^+^ in both the striola and extrastriola. In *Atoh1* overexpression mice, type I HCs lost the long and thin neck, and their nuclei were located at the same level as type II HCs. Meanwhile, these HCs lost markers of type I HCs (SPP1 and OCM), gained markers of type II HCs (SOX2 and ANXA4), had shorter bundles and a discontinuous calyx, and thus were named type II-like HCs. **B** In the cochlea of WT mice, IHCs expressed VGLUT3, while OHCs expressed Prestin. In the *Atoh1* overexpression mice, VGLUT3 was not expressed in IHCs, and the staining of Prestin in OHCs was weak and uneven, with OHCs becoming shorter in length and larger in diameter. With *Atoh1* constitutive overexpression HCs were progressively lost from the neonatal (P7) to adult (P35) stage

To further investigate the effects of permanent *Atoh1* expression on the maturation and survival of HCs, we developed an inducible system to overexpress *Atoh1* in HCs at specific developmental stages. We found that constitutive overexpression of *Atoh1* impaired vestibular function due to the disturbed differentiation of vestibular HC subtypes (Fig. 1A). Meanwhile, *Atoh1* overexpression in the cochlea at different developmental stages blocked the postnatal development of HCs and led to progressive HC loss and subsequent hearing impairment (Fig. 1B). Our study suggests that the proper duration of *Atoh1* expression is essential for the differentiation of HC subtypes, HC survival, and HC function in the inner ear.

## Results

### Overexpression of *Atoh1* in vestibular HCs impaired vestibular function

**Fig. 2:**
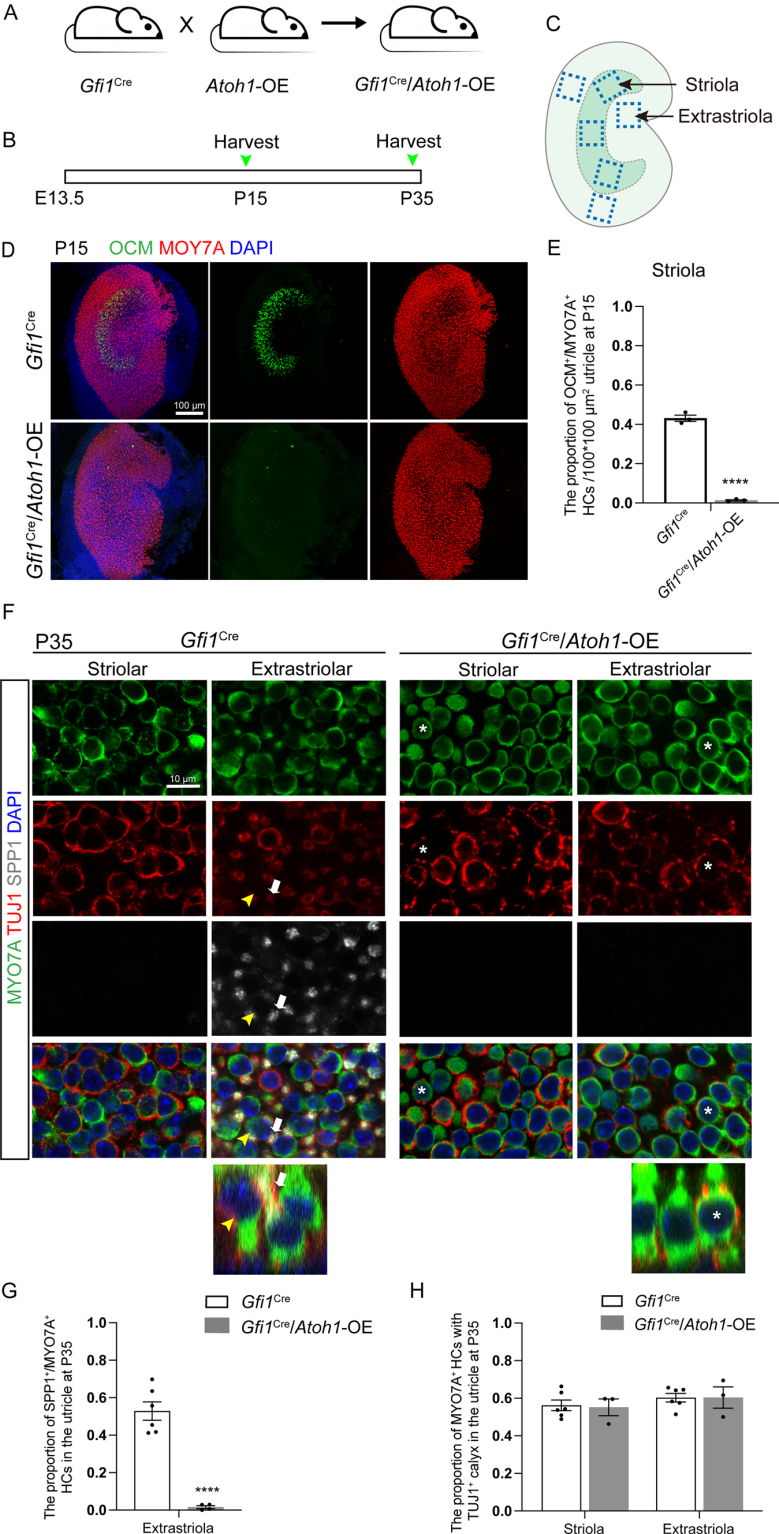
*Atoh1* overexpression interfered with type I HC differentiation in the striola and extrastriola of the utricle. **A** *Gfi1*^Cre^ mice were crossed with CAG-loxP-stop-loxP-*Atoh1*-HA mice to generate *Gfi1*^Cre^/*Atoh1*-OE mice. **B** The experimental protocol. *Atoh1* overexpression was induced from E13.5 in the vestibule, and mice were sacrificed at P15 and P35. **C** Schematic showing the utricle and its different zones, including the striola and the extrastriola. **D** OCM-labeled type I HCs in the striolar region of the utricle in control mice at P15. Few OCM^+^/MYO7A^+^ HCs were detected in the striolar region of the utricle of *Gfi1*^Cre^/*Atoh1*-OE mice at P15. **E** Quantification and comparison of OCM^+^/MYO7A^+^ HCs in the utricle of control and *Gfi1*^Cre^/*Atoh1*-OE mice at P15. **F** Co-immunolabeling of MYO7A, TUJ1, and SPP1 in the striola and extrastriola of the utricle at P35. No HCs were labeled by SPP1 in the striola. SPP1 labeled Type I HCs in the extrastriola of control mice, with full calyxes (white arrow). The yellow arrowhead shows SPP1^−^/MYO7a^+^ type II HCs with no calyx. Nearly no SPP1 was detected in the extrastriola of the *Gfi1*^Cre^/*Atoh1*-OE utricle. * indicates SPP1^−^/MYO7A^+^ HCs with partial calyxes in the striola and extrastriola. **G** Comparison of the proportion of SPP1^+^/MYO7A^+^ HCs in the extrastriola of the utricle at P35. **H** Comparison of the proportion of MYO7A^+^ HCs with TUJ1^+^ calyxes in the utricle at P35. Scale bars: 100 μm in **D**, 10 μm in **F**. Data in **E**, **G**, and **H** are presented as the mean ± SEM. Unpaired *t*-test in **E** and **G**. Two-way ANOVA in **H**. ****p < 0.0001

*Atoh1* expression in the vestibular system starts at E12.5 and is essential to vestibular HC differentiation and regeneration [7, 23, 24]. In the utricle, deletion of *Atoh1* at E14.5 or E16.5 did not cause cell death but led to the obstruction of MYO7A expression and defective stereocilium formation [19]. To investigate the effect of *Atoh1* overexpression on the epithelium of vestibular organs, we crossed *Gfi1*^Cre^ mice with CAG-loxP-stop-loxP-*Atoh1*-HA mice (Fig. 2A). *Gfi1* is a nuclear zinc-finger protein expressed soon after HC differentiation in the inner ear [35], and the expression of *Gfi1* is first detected at E13.5 in the vestibule and at E15.5 in the cochlea [36]. We used *Gfi1*^Cre^/CAG-loxP-stop-loxP-*Atoh1*-HA (*Gfi1*^Cre^/*Atoh1*-OE) mice to increase the expression of *Atoh1* in *Gfi1*^+^ cells in the vestibular organs, and mice lacking the *Atoh1*-OE allele were used as controls.

First, we assessed the gross vestibular function of *Gfi1*^Cre^/*Atoh1*-OE mice. We observed spontaneous head bobbing from P12–P13 in *Gfi1*^Cre^/*Atoh1*-OE mice, which might indicate vestibular dysfunction. When we held the P35 mice by their tails and slowly lowered them onto the ground, the control mice could extend their forelimbs and reach the horizontal surface, while most *Gfi1*^Cre^/*Atoh1*-OE mice could only bend their bodies ventrally (Fig. S1B, C) [37]. Then we performed a swimming test at P35, and the general vestibular function of both control and *Gfi1*^Cre^/*Atoh1*-OE mice were scored [37]. Control mice from the same litter engaged in normal swimming behaviors in which the tail propelled their movement through the water. In contrast, *Gfi1*^Cre^/*Atoh1*-OE mice showed unbalanced swimming behavior, in which the mice could not balance their heads or backs, or the mice would float immobile (Fig. S1D). The average score was 1.33 ± 0.33 for *Gfi1*^Cre^/*Atoh1*-OE mice at P35, which was significantly higher than the control mice (Fig. S1E). Next, we used a binocular video-oculography-based vestibular function testing system to quantitatively evaluate the vestibular-ocular reflex. The horizontal angular vestibulo-ocular reflexes (HAVOR) test was performed at P35 to detect the function of the horizontal cristae [38, 39]. Compared with control mice, the HAVOR gain of *Gfi1*^Cre^/*Atoh1*-OE mice decreased significantly at all frequencies (Fig. S1F). To observe the function of macular organs, an off-vertical axis rotation (OVAR) test was conducted. The eye movement amplitude in the OVAR test was significantly reduced in *Gfi1*^Cre^/*Atoh1*-OE mice (Fig. S1G). Together, these results indicate the gross vestibular function was impaired in the *Atoh1*-overexpressing mice. Meanwhile, HAVOR and OVAR responses were abnormal, which indicated that overexpression of *Atoh1* in vestibular HCs brought about the dysfunction of the vestibular-ocular reflex.

### Overexpression of *Atoh1* obstructed subtype differentiation in HCs

To assess the morphological features of vestibular HCs in *Gfi1*^Cre^/*Atoh1*-OE mice, we labeled HCs with MYO7A. We counted the numbers of MYO7A^+^ HCs in the striolar and extrastriolar regions in the utricles and saccules and the HC number in the central and peripheral crista zones separately at P15 and P35. The HC numbers were comparable in the vestibular organs of *Gfi1*^Cre^/*Atoh1*-OE mice and control mice at P15 and P35 (Fig. S2C). To further investigate the effect of *Atoh1* overexpression on the HCs, we observed the subtype of HCs in the vestibular organs.

Vestibular HCs come in two subtypes, type I and type II, which can be distinguished by molecular markers, the morphology of the cell body, physiological features, and nerve terminals [40–43]. Type I HCs are flask-shaped with a narrow neck, have relatively long stereocilia bundles, and are innervated by calyceal endings. In contrast, type II HCs are cylindrical and have shorter stereocilia and bouton synapses (Fig. 1A). To detect the effect of *Atoh1* overexpression on the subtype differentiation of HCs, we assessed the molecular, morphologic, and synaptic features representing the specialization of HC subtypes. First, we stained type I HCs with Oncomodulin (OCM), which is highly enriched in type I HCs in the striolar region [44, 45]. In the control mice, many OCM^+^/MYO7A^+^ type I HCs were observed in the striolar region of the utricle. In contrast, few OCM^+^/MYO7A^+^ HCs were identified in any sensory epithelia of the utricle in *Gfi1*^Cre^/*Atoh1*-OE mice (Fig. 2D, E, Fig. S3, A–C). We stained Secreted phosphoprotein 1 (SPP1) to label type I HCs in the extrastriolar region [42]. In the utricles of control mice, we detected SPP1^+^/MYO7A^+^ HCs in the extrastriolar region, while we found nearly no SPP1^+^/MYO7A ^+^ HCs in the *Gfi1*^Cre^/*Atoh1*-OE utricles (Fig. 2F, G). Similar results were found in the saccule and crista (Figs. S2 A, B, D, E and S3 A–G).

**Fig. 3:**
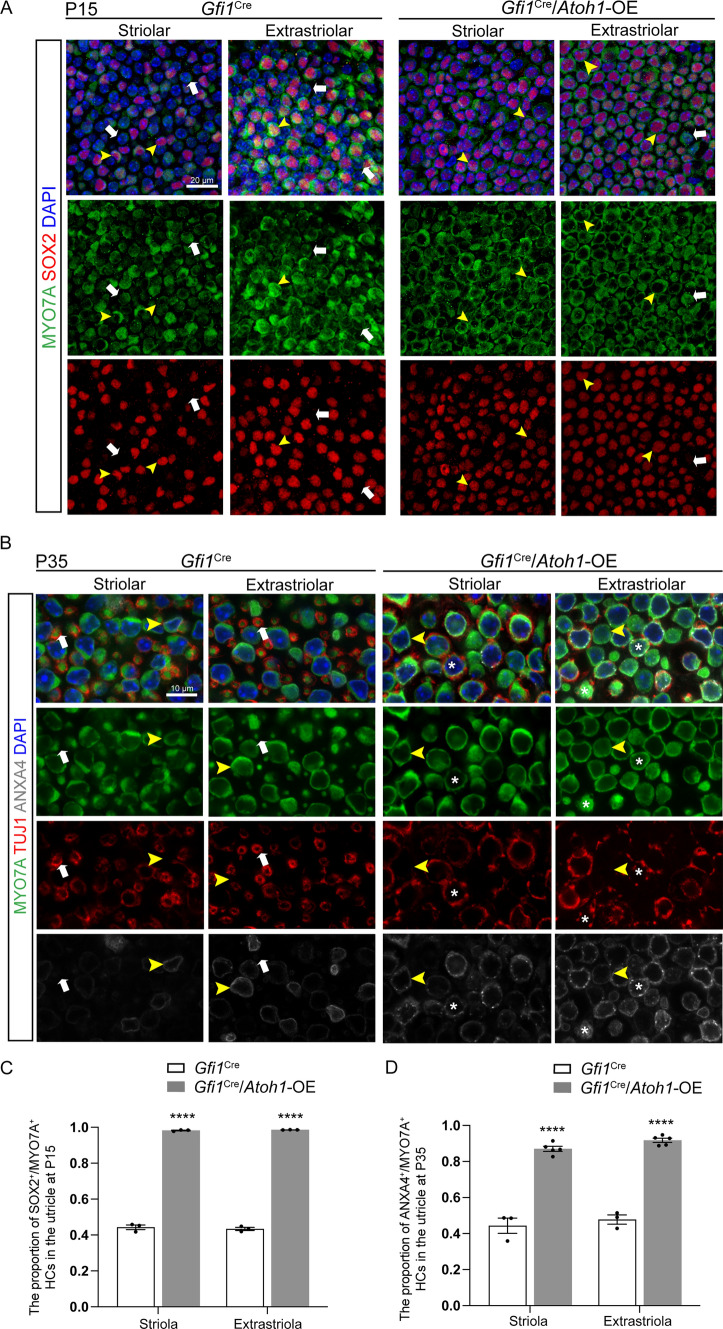
*Atoh1* overexpression promoted the expression of type II HC markers in the utricle. **A** Co-immunolabeling of MYO7A and SOX2. The white arrowhead indicates SOX2^−^/MYO7A^+^ type I HCs, while the yellow arrowhead indicates SOX2^+^/MYO7A^+^ type II HCs. In the utricle of *Gfi1*^Cre^/*Atoh1*-OE mice, few SOX2^−^/MYO7A^+^ type I HCs were detected. **B** Co-immunolabeling of MYO7A and ANXA4. The white arrow indicates ANXA4^−^/MYO7A^+^ type I HCs with a full calyx, while the yellow arrowhead indicates ANXA4^+^/MYO7A^+^ type II HCs with no calyx. More ANXA4^+^/MYO7A^+^ HCs were found in the *Gfi1*^Cre^/*Atoh1*-OE utricle. * marks ANXA4^+^/MYO7A^+^ HCs with a partial calyx. **C** Quantification and comparison of the proportion of SOX2^+^/MYO7A^+^ HCs in the utricle at P15. **D** Quantification and comparison of the  proportion of ANXA4^+^/MYO7A^+^ HCs in the utricle at P35. Scale bars: 20 μm in **A**, 10 μm in **B**. Data in **C** and **D** are presented as the mean ± SEM. Two-way ANOVA. ****p < 0.0001

Next, we detected the markers of type II HCs. *Sox2*, which is expressed in all developing HCs but persists only in type II HCs and SCs in the mature vestibular epithelia [42], is a marker to distinguish type I and type II HCs. We compared the proportion of SOX2^+^/MYO7A^+^ type II HCs from the striolar and extrastriolar regions of the utricle. In control mice, only 44.33 ± 1.28% of the HCs in the striolar region and 43.42 ± 0.84% of the HCs in the extrastriolar region were SOX2^+^/MYO7A^+^ type II HCs. In contrast, the proportion of SOX2^+^/MYO7A^+^ type II HCs increased significantly to 98.27 ± 0.23% in the striolar region and to 98.60 ± 0.04% in the extrastriolar region of the utricles from *Gfi1*^Cre^/*Atoh1*-OE mice (Fig. 3A, yellow arrowhead, Fig. 3C). We next observed type II HCs by staining for Annexin A4 (ANXA4), another molecular marker of type II HCs [42, 44–47]. Compared with control mice, the numbers of ANXA4^+^/MYO7A^+^ type II HCs in the *Gfi1*^Cre^/*Atoh1*-OE utricles were increased significantly in both the striolar and extrastriolar regions (Fig. 3B, D). Increased ratios of HCs with type II markers were also observed in the saccule and crista in *Gfi1*^Cre^/*Atoh1*-OE mice (Fig. S4A–H). These data suggested that *Atoh1* overexpression caused type I HCs to lose their markers SPP1 in the extrastriola and OCM in the striola and to gain the type II markers SOX2 and ANXA4 (Fig. 1A).

### *Atoh1* overexpression disrupted the development of calyx nerve terminals in type I HCs

We next focused on the nerve innervation and nerve terminals of *Atoh1* overexpression mice. Calyceal afferent nerve terminals are well-formed in all regions of the utricles during the first postnatal week [48–51]. Tuj1 staining results showed no significant difference in the density of nerve fibers in the vestibular organs between control and *Gfi1*^Cre^/*Atoh1*-OE mice at P35 (Fig. S8D). In the mature utricles of control mice, type I HCs were OCM^+^/ANXA4^−^/MYO7A^+^ in the striolar region and SPP1^+^/ANXA4^−^/MYO7A^+^ in the extrastriolar region, and they were enveloped with a well-developed full calyx (Figs. 2F, 3B). A total of 56.20 ± 2.80% of the HCs in the striolar region and 60.30 ± 2.20% of the HCs in the extrastriolar region of control utricles were surrounded with TUJ1^+^ neural terminals, while 55.20 ± 4.40% of the HCs in the striolar region and 60.40 ± 5.70% of the HCs in the extrastriolar region were surrounded in the *Gfi1*^Cre^/*Atoh1*-OE utricles. The ratios of HCs surrounded by TUJ1^+^ endings showed no significant difference between the two groups (Fig. 2H). In the control group, continuous TUJ1 signal enveloped the entire cell body of type I HCs at the level of the nucleus and extended along the cell’s neck (Figs. 2F, 3B, white arrow). However, in the *Gfi1*^Cre^/*Atoh1*-OE utricles, type I HCs that gained type II markers were surrounded by a discontinuous TUJ1^+^ calyx or were contacted with several thin neuronal processes but with no enclosed calyx (Figs. 2F, 3B, HCs labeled with “*”). This suggested that although type I HCs lost their markers and gained type II markers when *Atoh1* was overexpressed, these cells were not fully differentiated into type II HCs (Fig. 1A), and these are referred to as type II-like HCs in this study.

### *Atoh1* overexpression altered the morphology of HCs and interfered with the development of stereocilia bundles

We next observed the cell arrangement and morphology of HCs in the utricle. In the extrastriola of the control utricle, many type I HCs had nuclei located deep in the epithelium, just above the SC nuclei (Fig. 2F, white arrows). In contrast, type II HCs had nuclei closer to the lumen (Fig. 2F, yellow arrowheads). In the *Gfi1*^Cre^/*Atoh1*-OE utricle, almost all of the HC nuclei were located at the same level, while type II-like HCs lost the long and thin “neck” that is a feature of type I HCs (Figs. 1A, 2F, 3B, see the HCs labeled with “*”).

**Fig. 4:**
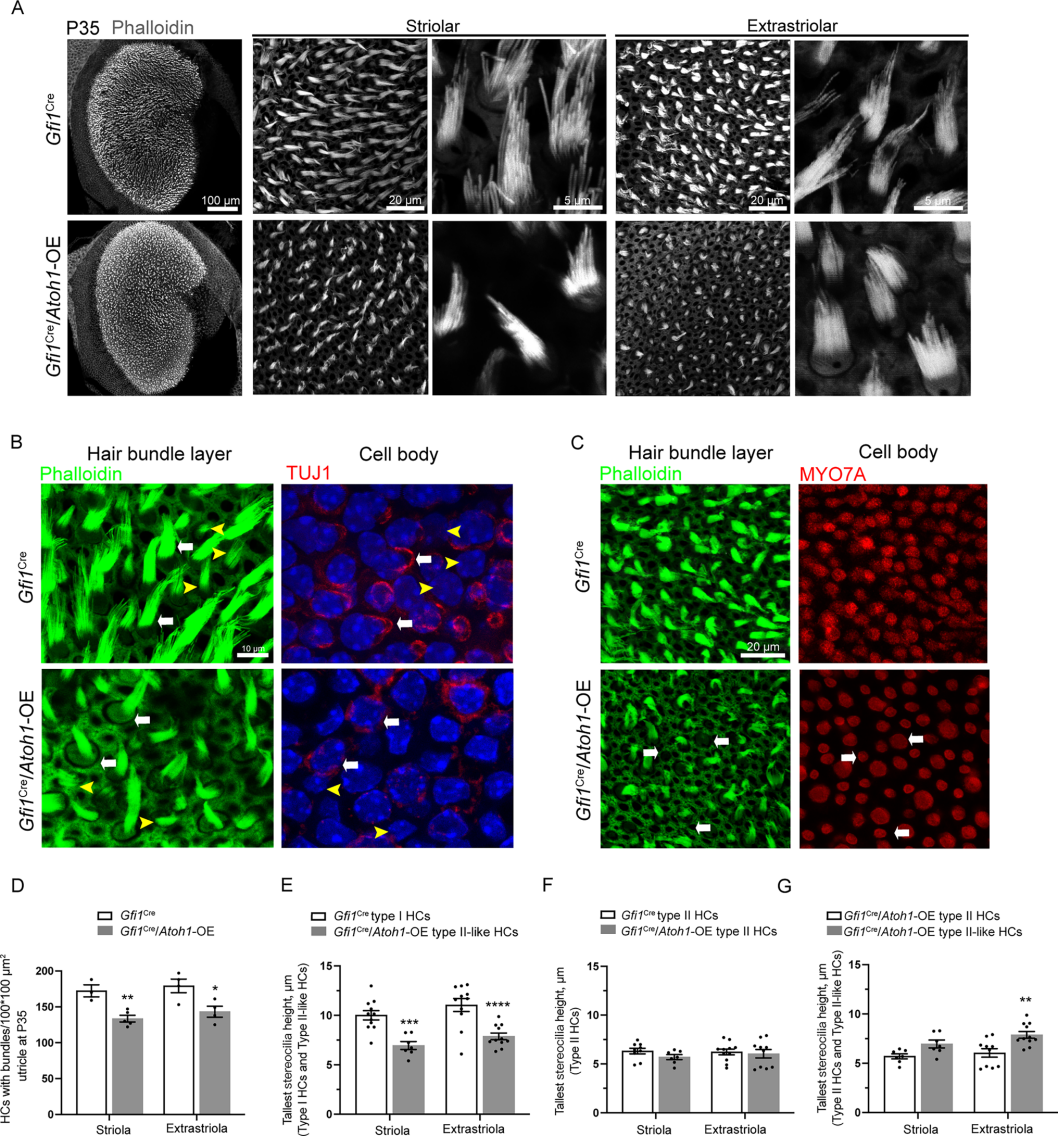
*Atoh1* overexpression disturbed the development of stereocilia bundles in the utricle. **A** Phalloidin-labeled stereocilia bundles at P35. In *Gfi1*^Cre^/*Atoh1*-OE mice, bundles became fewer and shorter in both the striola and extrastriola. **B** HCs labeled by Phalloidin in bundles and TUJ1 in the calyx. Bundles in HCs with a full calyx (white arrow) were longer than those in HCs without a calyx (yellow arrowhead) in control mice. Bundles in HCs with discontinuous calyxes (white arrow) in *Gfi1*^Cre^/*Atoh1*-OE mice were still longer than those in HCs without calyxes (yellow arrowhead) in control mice. **C** HCs labeled with Phalloidin and MYO7A. In the utricle of *Gfi1*^Cre^/*Atoh1*-OE mice, some HCs lost their stereocilia bundles (white arrow). **D** Quantification of HCs with stereocilia bundles in the striola and extrastriola of the utricle at P35. **E** Comparison of the length of the tallest bundles between type I HCs in the control group and type II-like HCs in the *Gfi1*^Cre^/*Atoh1*-OE group. **F** Comparison of the length of the tallest bundles between type II HCs in the control group and *Gfi1*^Cre^/*Atoh1*-OE group. **G** Comparison of the length of the tallest bundles between type II HCs in the control group and type II-like HCs in the *Gfi1*^Cre^/*Atoh1*-OE group. Scale bars: 100, 20, and 5 μm in **A**, 10 μm in **B**, and 20 μm in **C**. Data in **D**–**G** are presented as the mean ± SEM. Two-way ANOVA. *p < 0.05, **p < 0.01, ***p < 0.001, ****p < 0.0001

We next assessed the stereocilia bundles, one of the markers of HC maturation, by staining the actin-rich stereocilia with fluorescently labeled phalloidin. We observed a significant loss of bundles in *Gfi1*^Cre^/*Atoh1*-OE utricles at P35 (Fig. 4A, C, D), and the remaining stereocilia bundles were significantly shorter in the utricular HCs in *Gfi1*^Cre^/*Atoh1*-OE mice at P35 (Fig. 4A). For an accurate assessment, we measured and compared the bundle length between type I HCs with continuous calyxes in control mice and type II-like HCs with discontinuous TUJ1^+^ calyxes in *Gfi1*^Cre^/*Atoh1*-OE mice, and we found that the length of the tallest stereocilia bundles was decreased significantly in type II-like HCs in the *Gfi1*^Cre^/*Atoh1*-OE utricles (Fig. 4B, E). We found no significant difference in the length of the tallest stereocilia bundles between type II HCs in the control utricles and type II HCs in the *Gfi1*^Cre^/*Atoh1*-OE utricles (Fig. 4B, F). We next compared the tallest stereocilia bundle length between type II HCs in the control utricles and type II-like HCs in the *Gfi1*^Cre^/*Atoh1*-OE utricles. Although shorter than type I HC bundles in the control utricle, the bundles of type II-like HCs were significantly longer than those of type II HCs (Fig. 4B, G).

Our findings suggest that constitutive *Atoh1* expression interfered with the differentiation of type I HCs and calyceal nerve terminal formation and resulted in shorter hair bundle structures, which led to the dysfunction of the vestibular system without HC loss (Fig. 1A).

### Overexpression of *Atoh1* in the cochlea did not affect the fate determination of HCs

**Fig. 5:**
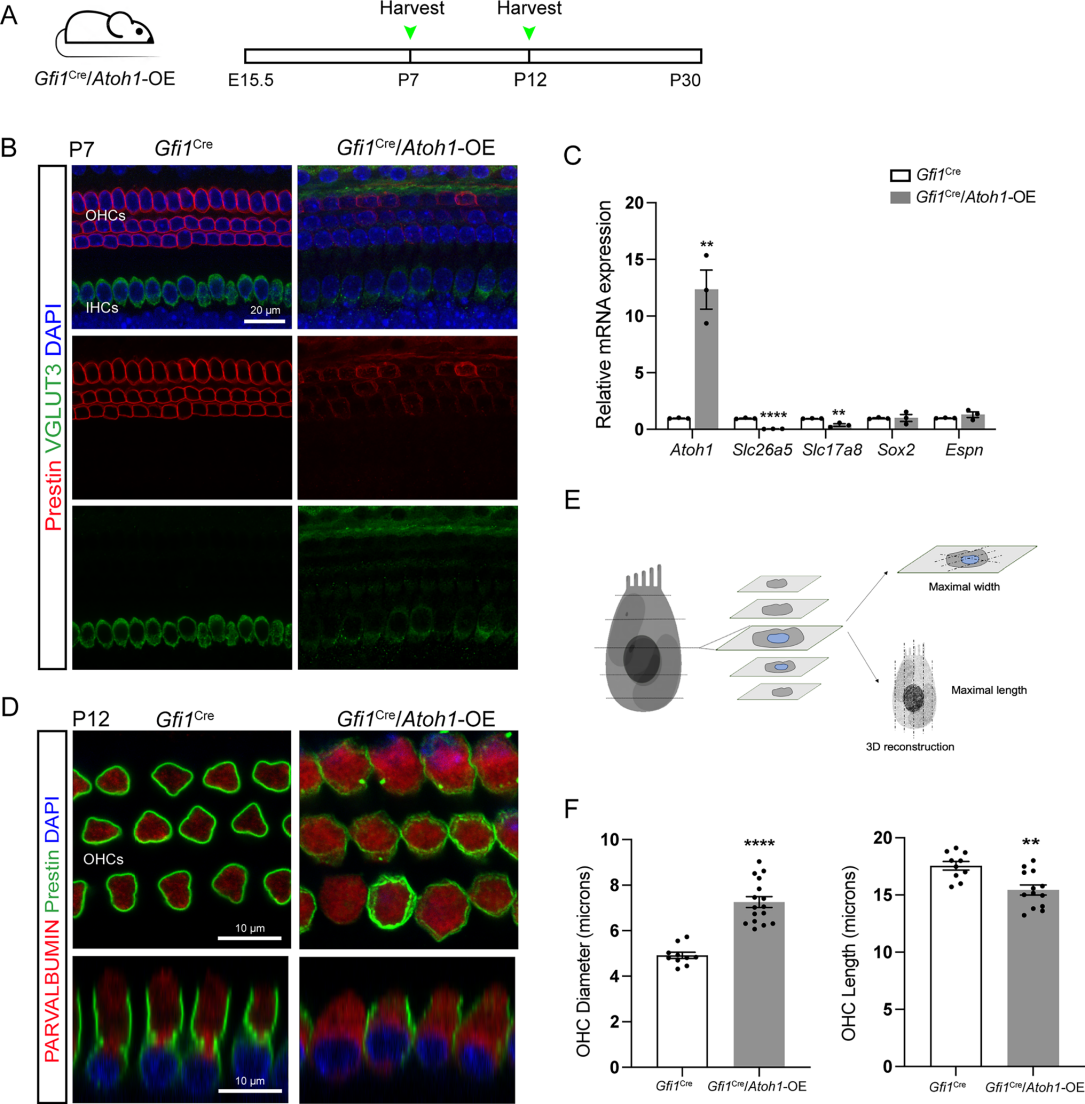
Permanent *Atoh1* overexpression in the cochlea from the embryonic stage led to HC development disorders. **A** The experimental protocol. *Atoh1* overexpression was induced from E15.5, and mice were sacrificed at P7 or P12. **B** The OHCs were labeled by Prestin, while VGLUT3 labeled the IHCs in the control cochlea at P7. In the *Gfi1*^Cre^/*Atoh1*-OE mouse cochlea, the staining of Prestin and VGLUT3 was weak and uneven. **C** The relative mRNA expression levels of *Atoh1*, *Myo7a*, *Slc26a5*, *Slc17a8*, *Sox2*, *Pou4f3*, and *Espn* at P7. **D** The staining of Prestin and VGLUT3 was weak and uneven in the *Gfi1*^Cre^/*Atoh1*-OE cochleae at P12. **E** Schematic illustrating the measurement protocol. **F** OHC diameter and length of control and *Gfi1*^Cre^/*Atoh1*-OE mice at P12. Scale bars: 10 μm in the last figure of K and 20 μm in the other figures. Data in M are presented as the mean ± SEM. Unpaired *t*-test. **p < 0.01, ****p < 0.0001

*Atoh1* expression, which begins between E12.5 and E14.5 in the mouse cochlear epithelium, is necessary to specify HCs in the inner ear, and the organ of Corti of the *Atoh1*-null mouse fails to generate HCs [7, 10]. The sufficient level and duration of *Atoh1* expression are also crucial for the differentiation of HCs. A previous study showed that when *Atoh1* was knocked out in mice during E15.5–E17.5, different degrees of HC loss were observed in the cochlea at P0 [17–19]. To test the effect of *Atoh1* overexpression on cell fate determination and the survival of cochlear HCs during the embryonic stage, we used *Gfi1*^Cre^/*Atoh1*-OE mice in which *Atoh1* was constitutively overexpressed in cochlear HCs from E15.5. We performed immunohistochemistry on whole-mount cochleae at P2 and P7 (Fig. S5 A) and found no HC death in either control or *Gfi1*^Cre^/*Atoh1*-OE cochleae (Fig. S5B–G). In *Atoh1*-OE cochleae at P7, the morphology of cochlear HCs looked normal, with neatly arranged a single row of inner hair cells (IHCs) and three rows of outer hair cells (OHCs), and the cochlear HCs were SOX2 negative (Fig. S5E). We next investigated the effect of *Atoh1* overexpression on hair bundle structure by staining for phalloidin. The staining results showed typical hair bundle structures in *Gfi1*^Cre^/*Atoh1*-OE mice at P2 and P7, and these were organized into a V-formation in OHCs and were aligned at the apical surface of IHCs, suggesting no defects in planar cell polarity (Fig. S5C, D). Meanwhile, qPCR results showed no difference in the transcriptional level of *Espn*, which encodes the actin-crosslinking protein ESPIN that is essential for HC bundle formation, between control and *Gfi1*^Cre^/*Atoh1*-OE cochleae at P7 (Fig. 5C).

### Overexpression of *Atoh1* delayed the maturation of young HCs in the cochlea

The development of HCs in the mouse is a protracted process, extending into the postnatal period. Previous studies have shown that *Atoh1* expression is gradually downregulated in cochlear HCs during postnatal maturation and falls to a low level at P7 [15, 16] when cochlear HCs begin to display the morphological and electrophysiological features of mature cochlear HCs. We therefore asked whether *Atoh1* overexpression would disturb the maturation of HCs in the cochlea. We focused on two markers, vesicular glutamate transporter 3 (VGLUT3, encoded by *Slc17a8*) and Prestin (encoded by *Slc26a5*), that are necessary for the normal function of HCs. VGLUT3 is expressed explicitly in IHCs and accumulates glutamate in the presynaptic vesicles of the IHCs, and the loss of VGLUT3 causes deafness. VGLUT3 is turned on primarily in IHCs at E16.5, increases at P3, and is maintained at a steady level thereafter [52–57]. Prestin is a motor protein responsible for the fast electromotility of OHCs [58–60]. Prestin can be detected in the lateral wall of OHCs as early as P0, and the intensity of its labeling reaches adult levels at P9–P11 [59–61]. We found that protein levels of Prestin and VGLUT3 were both lower in the cochlear HCs in *Gfi1*^Cre^/*Atoh1*-OE mice at P7 when compared to control mice (Fig. 5B). The downregulation of *Slc26a5* and *Slc17a8* mRNA was further confirmed by qPCR in the cochlear epithelia from P7 *Gfi1*^Cre^/*Atoh1*-OE mice (Fig. 5C). After another 5 days, the expression of Prestin was slightly modified in OHCs in *Gfi1*^Cre^/*Atoh1*-OE mice. Still, its expression was distributed unevenly and divergently on the membrane of OHCs, which was significantly different from the linear and uniform distribution pattern in the OHCs from control mice (Fig. 5D). Meanwhile, the OHCs of *Gfi1*^Cre^/*Atoh1*-OE cochleae became bigger in diameter and shorter in length (Fig. 5D–F). Figure 1F shows the significant difference in the diameter and length of OHCs between control and *Gfi1*^Cre^/*Atoh1*-OE mice.

SOX2 can be used as a marker for immature HCs, and it is expressed in both the SCs and HCs at E15.5 and then gradually diminishes in cochlear HCs [8, 62, 63]. SOX2 can still be detected in the cochlear HCs of neonatal mice but becomes undetectable at P7 [62, 64]. We further investigated the expression of SOX2 in the cochlear HCs from P7 *Gfi1*^Cre^/*Atoh1*-OE mice, and no SOX2^+^ HCs were observed (Fig. S5E). Meanwhile, there was no difference in the transcriptional level of *Sox2* between the control and *Gfi1*^Cre^/*Atoh1*-OE cochleae at P7 (Fig. 5C).

To further verify the effect of long-term *Atoh1* expression in HCs that have already undergone primary differentiation in the cochlea at the postnatal stage, we generated *Atoh1*^CreER^-CAG-loxP-stop-loxP-*Atoh1*-HA (*Atoh1*^CreER^/*Atoh1*-OE) mice (Fig. S6A) in which constitutive activation of *Atoh1* in *Atoh1*^+^ cells was induced by tamoxifen administration at P2 [13, 65]. Cochlear sensory epithelial tissues were analyzed at P7 (Fig. S6B), and mice lacking the *Atoh1*-OE allele were used as controls. Consistent with the results obtained from the *Gfi1*^Cre^/*Atoh1*-OE cochleae, no HC loss was observed in the cochleae (Fig. S6C, D); however, the expression levels of Prestin and VGLUT3 were down-regulated and distributed unevenly in the OHCs and IHCs from P7 *Atoh1*^CreER^/*Atoh1*-OE mice (Fig. S6D). The downregulation of *Slc26a5* and *Slc17a8* mRNA was further confirmed by qPCR in cochlear epithelial samples from P7 *Atoh1*^CreER^/*Atoh1*-OE mice, indicating that *Atoh1* overexpression inhibited the maturation of cochlear HCs (Fig. S6E, Fig. 1B).

### Overexpression of *Atoh1* changed the expression pattern of Prestin in relatively mature OHCs in the cochlea

Because we showed that overexpression of *Atoh1* in native cochlear HCs could inhibit the maturation of OHCs and IHCs during the first week after birth, we further investigated the effects of constitutive expression of *Atoh1* on relatively mature OHCs after P7. We generated the transgenic mouse model *Prestin*^CreER^/CAG-loxP-stop-loxP-*Atoh1*-HA (*Prestin*^CreER^/*Atoh1*-OE) [59] in which *Atoh1* is overexpressed in *Prestin*^+^ OHCs after tamoxifen administration at P7 (Fig. S6F, G). Mice lacking the *Atoh1*-OE allele were used as controls. We also observed that Prestin was downregulated in the surviving OHCs (note the arrowhead pointing to MYO7A^+^ HCs with weak Prestin staining) and was present in an uneven and divergent pattern on the membrane of OHCs from the *Prestin*^CreER^/*Atoh1-*OE cochleae at P25 (Fig. S6H). There was a significant difference between the control and *Prestin*^CreER^/*Atoh1*OE in the diameter and length of OHCs, similar to the phenomenon we observed in the OHCs from *Gfi1*^Cre^/*Atoh1*-OE mice (Fig. S6I).

### Constitutive expression of *Atoh1* led to progressive HC loss and hearing impairment in the cochlea

**Fig. 6:**
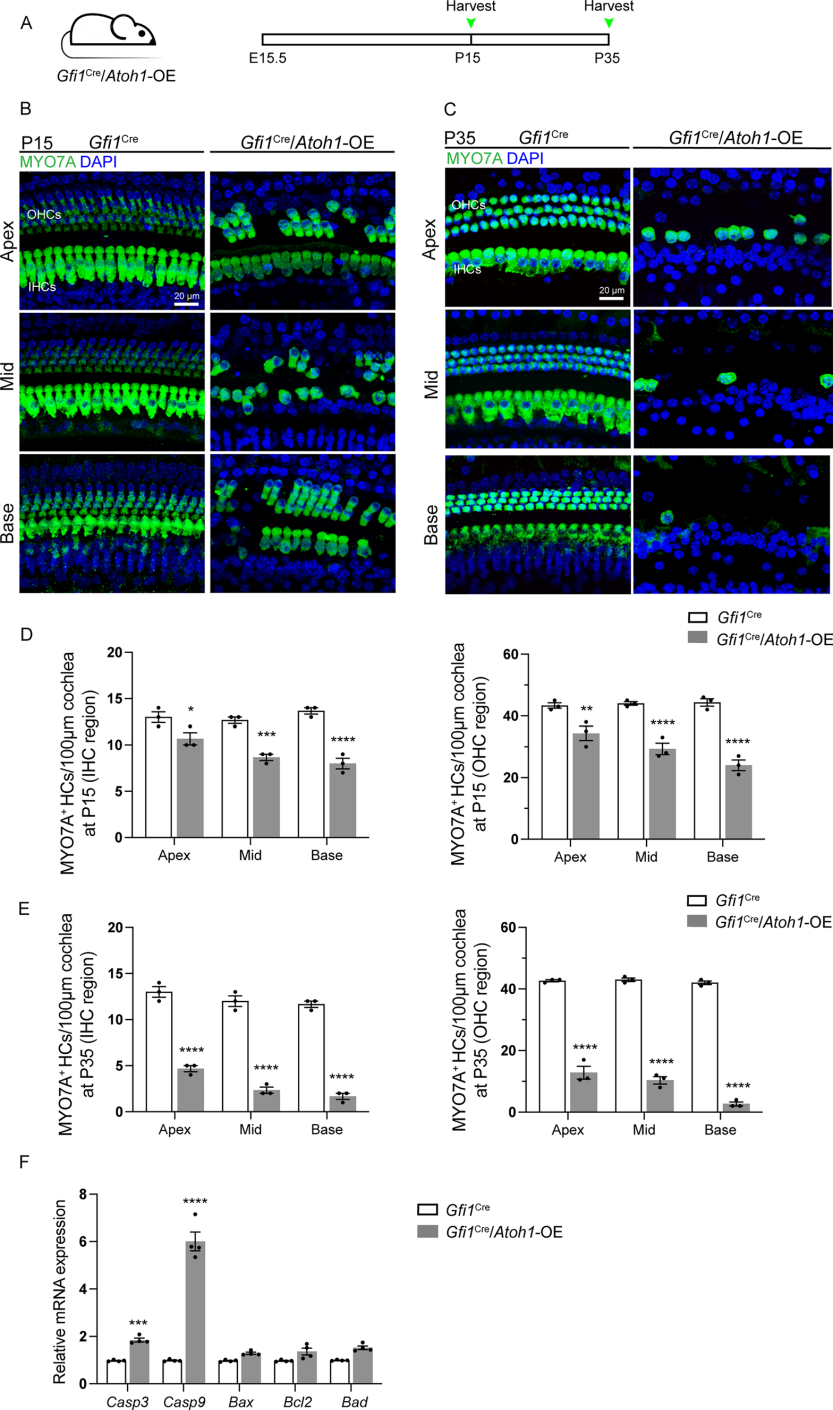
Overexpression of *Atoh1* in cochlear HCs in embryonic and neonatal mice led to progressive HC loss. **A** The experimental protocol. *Atoh1* overexpression was induced from E15.5, and mice were sacrificed at P15 and P35. **B** No HC loss was observed in control cochleae, but obvious OHC and IHC loss was seen in *Gfi1*^Cre^/*Atoh1*-OE cochleae at P15. **C** No HC loss was detected in control cochleae at P35, and only a few HCs remained in the *Gfi1*^Cre^/*Atoh1*-OE cochleae at P35. **D** The number of OHCs and IHCs in the *Gfi1*^Cre^/*Atoh1*-OE and control cochleae at P15. **E** The number of OHCs and IHCs in the *Gfi1*^Cre^/*Atoh1*-OE and control cochleae at P35. **F** Relative mRNA expression of apoptosis-related genes in the cochlea at P15. Scale bars: 20 μm. Data are presented as the mean ± SEM. Two-way ANOVA. *p < 0.1, **p < 0.01, ***p < 0.001, ****p < 0.0001

Although we saw no loss of HCs when we examined mice at P7, we did observe HC loss at later ages induced by the constitutive expression of *Atoh1* at different developmental stages. In *Gfi1*^Cre^/*Atoh1*-OE mice, in which *Atoh1* is overexpressed in the differentiating cochlear HCs, significant HC loss was observed in both IHCs and OHCs at P15 (Fig. 6B). The number of HCs decreased significantly compared with the control mice (Fig. 6D). At 35 days after birth, almost no OHCs and only a few IHCs were seen in the cochleae of *Gfi1*^Cre^/*Atoh1*-OE mice (Fig. 6C, E).

Meanwhile, the overexpression of *Atoh1* in P2 *Atoh1*^CreER^/*Atoh1*-OE mice also induced scattered OHC loss from the apical to the basal turn of the cochlea at P11 (Fig. S7A, B). The numbers of remaining OHCs in the apical and basal turns differed significantly between the control and *Atoh1*^CreER^/*Atoh1*-OE mice (Fig. S7C). However, because the *Atoh1*^CreER^/*Atoh1*-OE mice did not survive after P11, we could not follow the status of HCs in the cochlea at older ages.

For the effects of constitutive *Atoh1* expression on relatively mature OHCs, tamoxifen was administered to *Prestin*^CreER^/*Atoh1*-OE mice at P7, and the cochlear sensory epithelium was dissected out for immunohistochemistry staining at P21, P25, P35, and P53 (Fig. S7D). Hearing was evaluated through auditory brainstem response (ABR) at P25 and P35, which showed a 45–50 dB threshold shift at P25 and a 50–55 dB threshold shift at P35 in *Prestin*^CreER^/*Atoh1*-OE mice (Fig. S7E). In contrast, the control mice from the same litter had normal hearing thresholds. We observed scattered OHC loss from the apical to the basal turns in the cochlea at P21 and P25 and significant OHC loss at P35 and P53 (Fig. S7F) in *Prestin*^CreER^/*Atoh1*-OE mice. We counted the residual OHCs from P21, P25, P35, and P53 *Prestin*^CreER^/*Atoh1*-OE mice and found that the numbers decreased significantly with age (Fig. S7G). These results suggest that constitutive *Atoh1* expression in relatively mature HCs results in the death of HCs and subsequent hearing loss (Fig. 1B). Next, we observed the spiral ganglion neurons and the nerve fibers in the cochlea and found no obvious loss of nerve fibers or spiral ganglion neurons at P35 (Fig. S8A, C).

We next investigated the expression of apoptosis-related genes in control and *Gfi1*^Cre^/*Atoh1*-OE cochleae at P15. qPCR data showed that *Gfi1*^Cre^/*Atoh1*-OE mice had significantly higher expression of the pro-apoptosis genes *Casp3* and *Casp9* in the cochleae when compared to control mice (Fig. 6F), demonstrating that *Atoh1* overexpression induced caspase-mediated apoptosis.

## Discussion

Previous studies have shown that the upregulation of *Atoh1* can convert SCs into HCs in mouse auditory and vestibular organs in the inner ear of the neonatal and adult mouse in vivo [22, 23, 25, 30, 34, 66]. *Atoh1* has been the most critical gene for promoting the regeneration of HCs and restoring function in the inner ear. In previous studies, *Atoh1* was constitutively overexpressed to drive SCs to transdifferentiate into HCs using genetic tools or transgenic mouse models [23, 25, 29–31, 66]. However, in previous studies, no fully mature and functional HCs have been identified by overexpressing *Atoh1* alone in non-sensory cells, which might be due to insufficient signals for generating mature HCs or to side effects from the consistently high level of *Atoh1* [22, 23, 34, 66]. Here we describe in detail the impact of the permanent expression of *Atoh1* on the maturation and survival of HCs through overexpression of *Atoh1* in HCs at three different developmental stages in the mouse inner ear. Our results suggested that the proper duration of *Atoh1* expression is essential for the survival and function of HCs in the inner ear.

### *Atoh1* overexpression disrupts HC subtype differentiation in the vestibular organs

While spontaneous HC replacement does not occur in the cochlea, it happens to some degree in the mature mammalian vestibular epithelium, such as the utricle [3, 67–71]. In the damaged mature utricle, *Atoh1* overexpression enhances HC regeneration, resulting in more robust vestibular-evoked potential responses [23]. Regenerated HCs have been reported to be type II HCs [2, 14]. However, Sayyid et al. showed that spontaneously regenerated HCs in the utricle consist of HCs labeled separately by both type I and type II HC markers. The proportion of HCs marked by the type I marker increased over time. In the damaged utricle with *Atoh1* overexpression, the newly regenerated HCs are mainly composed of type II HCs instead of type I HCs [23]. In the utricle, *Atoh1* can be detected from E12.5 and is downregulated once HCs mature in type I HCs, but continues to be expressed in type II HCs [10, 14]. Our study found that overexpression of *Atoh1* in differentiating HCs in the vestibular organs resulted in a decrease in type I HCs and an increase in type II-like HCs. These type II-like HCs, which lost type I markers and gained type II markers, were surrounded by discontinuous calyx nerve terminals (Figs. 2, 3). These calyx nerve terminals were highly similar to the terminals of type I HCs in the utricle in the neonatal period [48], which suggested that *Atoh1* overexpression might restrict the development of type I HCs. The mechanism through which the two subtypes of vestibular HCs develop has not been fully elucidated. However, it is hypothesized that the two subtypes of vestibular HCs diverge from common HC progenitors and that type I HCs transit through a type II stage on their way to becoming “super-differentiated” HCs [41, 72–74].

### *Atoh1* overexpression impairs hair bundle maturation

Previous research showed that regenerated HCs driven by *Atoh1* overexpression displayed shorter or no hair bundles [23, 25]. In the vestibular organs, we identified fewer and shorter phalloidin-labeled hair bundle structures in type II-like HCs compared with bundles in type I HCs after the overexpression of *Atoh1* (Fig. 4). This suggested that incomplete bundle maturation might be due to permanent *Atoh1* expression. Previous studies have reported that the hair bundles of type II vestibular HCs are shorter and thinner than those of type I vestibular HCs [75–77]. We measured and compared the tallest length of bundles in type II HCs of control utricles and type II-like HCs of *Gfi1*^Cre^/*Atoh1*OE utricles, and the length of the bundles was significantly longer in the type II-like HCs of *Gfi1*^Cre^/*Atoh1*OE utricles (Fig. 4). Our results showed that overexpression of *Atoh1* disturbed the differentiation of type I vestibular HCs and HC bundle formation, resulting in vestibular system dysfunction. These results thus demonstrated that the permanent overexpression of *Atoh1* impedes the functional maturation of vestibular HCs. Whether Atoh1 overexpression-induced vestibular phenotypes change with increasing age needs further study in the future.

### *Atoh1* overexpression in the cochleae impairs the expression and function of Prestin in OHCs

We showed that overexpression of *Atoh1* during early developmental stages in the *Gfi1*^Cre^ mouse line (E15.5 in the cochlea) or during the neonatal stages in the *Atoh1*^CreER^ mouse line (P2) did not inhibit the primary development of cochlear HCs, which presented with relatively normal HC quantity, cellular shape, cellular polarity, and hair bundles during the first week after birth (Fig. S5). However, the expression of functional markers of IHCs and OHCs, VGLUT3 and Prestin, respectively, was significantly downregulated at the mRNA and protein levels at P7 (Fig. 5 and S6). Liu et al. analyzed *Gfi1*^Cre^/*Atoh1*-OE cochleae and found varied OHC loss and retention of Prestin expression in the residual OHCs at P14 of *Gfi1*^Cre^/*Atoh1*-OE cochleae [22], so they concluded that that the absence of Prestin in SC-derived new HCs was not caused by permanent *Atoh1* expression. However, in our study Prestin expression was slightly modified in OHCs in *Gfi1*^Cre^/*Atoh1*-OE mice at P12 compared to P7, but the distribution of Prestin was uneven and divergent across the membrane of OHCs (Fig. 5). Meanwhile, once *Atoh1* was re-activated in the relatively mature Prestin^+^ OHCs at P7, we observed the downregulation of Prestin in the OHCs, which presented as an uneven and divergent pattern across the membrane of OHCs (Fig. S6). Liberman et al. showed that the deletion of Prestin in mice results in the loss of electromotility, shortened OHCs, and loss of OHCs in the basal turn of the cochlea [78]. In another study, OHCs of mice expressing mutated Prestin were shorter than normal OHCs and died [79]. In our study, we found that OHCs became bigger in diameter and shorter in length in *Gfi1*^Cre^/*Atoh1*-OE mice at P12 (Fig. 5D–F) and in *Prestin*^CreER^/*Atoh1*-OE mice at P25 (Fig. S6). Our results coincided with those of mice that had mutated Prestin, which suggested that although there was expression of Prestin in the cochleae of *Gfi1*^Cre^/*Atoh1*-OE at P12, the function of HCs was still impaired. Thus, we concluded that the constitutive expression of *Atoh1* might inhibit the maturation of immature HCs and induce the degeneration of mature HCs during the development of the inner ear [31, 80].

### Prestin deficiency caused by *Atoh1* overexpression might result in OHC loss

Evidence has already shown that *Atoh1* is expressed during embryonic stages and is necessary for HC fate commitment [7, 10]. However, the expression of *Atoh1* declines dramatically within the first week after birth [15, 16], which suggests that *Atoh1* is not required for HC survival. In our experiments, no HC loss was detected in the cochleae of *Gfi1*^Cre^/*Atoh1*-OE or *Atoh1*^CreER^/*Atoh1*-OE mice at P7 (Fig. S5), indicating that *Atoh1* overexpression does not affect the initial differentiation of cochlear HCs. We observed scattered OHC loss in *Atoh1*^CreER^/*Atoh1*-OE cochleae at P11 and progressive HC loss in *Gfi1*^Cre^/*Atoh1*-OE mice around P15 (Fig. 6), accompanied by the decreased level and abnormal pattern of Prestin expression in the OHCs. These results coincided with the OHC loss pattern in the mice expressing mutated Prestin [79]. Prestin, a motor protein responsible for the fast electromotility of OHCs, can be detected in the lateral OHC membrane in neonatal mice, and its expression plateaus at about P10. *Prestin* knockout mice showed premature loss of OHCs in the basal quarter of the cochlea [78]. In mice with mutant Prestin, no missing OHCs were detected at P12, while progressive OHC loss was observed after P18 [79]. This implies that the inhibition and dysfunction of Prestin, which was caused by the consistently high level of *Atoh1*, impaired the survival of premature cochlear OHCs. On the other hand, Atoh1 overexpression might directly trigger apoptosis of cochlear HCs. It is possible that the downregulation of Prestin and VGLUT3 and changes in the diameter and length of OHCs might be a reflection of a protracted cell death process. Although we detected the expression changes of some HC maturation-related genes (like *Slc26a5* and *Slc17a8*) and several apoptosis-related genes (like *Casp3* and *Bcl2*), the expression of many other genes might also have been changed after consistent overexpression of *Atoh1*. A detailed analysis of the single-cell sequencing datasets of cochlear cells will better shed light on this issue.

### Time-restricted regulation of genes is vital for functional HC regeneration

The importance of critical genes’ timed and quantitative expression has been observed in other organs. For example, glial cell line-derived neurotrophic factor (GDNF) gene therapy has been reported to promote motor neuron survival and axon outgrowth. However, uncontrolled delivery of GDNF results in axon entrapment and impairs axon regeneration. On the other hand, the time-restricted expression of GDNF can significantly reduce nerve hypertrophy and axon entrapment and is sufficient to promote long-term motor neuron survival and facilitate the recovery of compound muscle action [81]. In addition, Krüppel-like factor 1 (*Klf1*) is a core cardiomyogenic trigger in zebrafish and is induced in adult zebrafish myocardium upon injury. However, zebrafish with constitutive *Klf1* expression exhibit heart failure-like symptoms and reduced survival, and only transient activation of *Klf1* yields mature myocardium without pathological dilation [82].

*Atoh1* is a vital gene for HC regeneration in the inner ear, and the first trial of gene therapy for deafness in humans based on delivering the recombinant adenovirus 5 vector containing the human cDNA of atonal transcription factor (*Hath1*), a homolog of *Atoh1*, is currently underway in the US (*NCT02132130*). The results of this trial have not been released yet. However, according to our results, the long-term expression of *Atoh1* by the adenovirus 5 vector might also be detrimental to the maturation and survival of the newly regenerated HCs in humans and impede the functional recovery of hearing. Thus, the proper duration of *Atoh1* expression should be considered when designing future gene therapy strategies for sensorineural deafness. Recently, we showed that co-expression of *Gfi1*, *Pou4f3*, and *Atoh1* could generate relatively more mature and functional IHCs than overexpression of *Atoh1* alone, which suggests that adding other crucial transcriptional factors for HCs might help overcome the limitation of *Atoh1* expression alone and might promote the further maturation of newly generated HCs from the direct trans-differentiation of SCs [31, 80].

## Materials and methods

### Animals

*Gfi1*^Cre^ mice [36] were generously provided by Lin Gan of the University of Rochester. *Atoh1*^CreERT2/+^(*Atoh1*^CreER^) [13] and *Prestin*^CreERT2/+^ (*Prestin*^CreER^) mice [59] were gifted by Liu Zhiyong of the Institute of Neuroscience, Chinese Academy of Sciences. CAG-loxP-stop-loxP-*Atoh1*-HA mice were kind gifts from St. Jude Children’s Research Hospital. Ai14 (Stock 007924) mice were purchased from The Jackson Laboratory. Transgenic mice were in the C57BL/6J background. To activate *Atoh1 *in vivo in HCs at different developmental stages, we bred CAG-loxP-stop-loxP-*Atoh1*-HA mice with mice expressing Cre under the control of different promoters. Tamoxifen diluted in corn oil was injected intraperitoneally once at P2 for *Atoh1*^CreER^ mice and at P7 for *Prestin*^CreER^ mice at 0.2 mg/g body weight. Male and female mice were used for all experiments, and all animal experiments were approved by the Institutional Animal Care and Use Committee of Fudan University.

### Vestibular function test

The tail-hanging reflex and swimming tests were evaluated at P30–35 to observe the vestibular function. The examination of tail-hanging reflex was performed by holding the mouse by the tail, lifting it to approximately 20 cm, and then descending to the starting point. Mice with normal behaviors would extend their forelimbs toward the ground, which scored 0, while the abnormal mice always ventrally bent their bodies, which scored 1. The swimming test was performed in a standard cage with at least 15 cm of water at 24–26 °C, which allowed the free swimming of mice. Mice were recorded on camera for a maximum of 1 min to assess their swimming ability on a scale of 0 to 3 [37]. The genotype of the tested mice was blinded to the investigator.

Accurate vestibular function was evaluated by a binocular video-oculography-based vestibular function testing system provided by Prof. Fangyi Chen’s team from the Southern University of Science and Technology. The HAVOR test was conducted to evaluate the function of the semicircular canals [38, 39]. Mice were fixed on the rotating platform with a noninvasive animal-immobility setup. The HAVOR test was performed under sinusoidal rotation with a peak velocity of 40º/s. Rotation stimuli at 0.5, 0.8, 1.0, 1.6, and 3.2 Hz were applied for no less than 90 s to collect adequate video data. The side cameras synchronously recorded eye movement under infrared illumination. The mouse’s pupil movement data were calibrated and calculated to obtain HAVOR gain values, defined as the amplitude ratio between response and stimulus [38]. To detect vestibular-ocular reflex responses derived from macular organs, we conducted the OVAR test, in which mice were fixed in the platform tilted 17° relative to the ground in a nose-down position and rotated at a constant velocity [39, 83]. The amplitude data of eye movement was recorded and compared.

### ABR recording

The hearing thresholds of the mice were examined with the ABR test. Animals were anesthetized with Dexdomitor (100 mg/kg) and Zoletil (25 mg/kg) and placed in a sound-attenuating chamber. Frequency-specific auditory responses were measured using the Tucker–Davis Technology system III, and all ABR tests were performed on mice at P25 and P35. Three mice were used in each experiment. Auditory thresholds were determined by decreasing the sound intensity of each stimulus from 90 to 20 dB in 5 dB steps at different frequencies (8, 16, 24, and 32 kHz) until the lowest sound intensity with reproducible and recognizable waveforms was detected. Thresholds were presented as the mean ± SEM and analyzed at individual frequencies using two-tailed *t* tests.

### RNA isolation and qPCR

After cochlear collection, the bone labyrinth and spiral ganglion were removed in phosphate-buffered saline (PBS). Cochlear samples from P7 mice did not include spiral ligaments and striate vascularis, but cochlear samples from P15 mice did include the spiral ligament and stria vascularis which were difficult to separate from the cochlear sensory epithelia. After isolation, three cochleae from each group were rapidly pooled in TRIzol (Thermo Fisher Scientific) to obtain the total RNA following the manufacturer’s instructions. cDNA was synthesized by reverse transcription using the 1st Strand cDNA Synthesis Kit (Takara) following the manufacturer’s protocol. qPCR was performed using a TB Green PrimeScript qPCR Kit (Takara) on a Bio-Rad 7500 detection system (Applied Biosystem). *Gapdh* was used as the housekeeping gene for endogenous reference. The quantification of relative gene expression, compared to *Gapdh*, was analyzed by the 2^−ΔΔCT^ method. Primer sequences are listed in Supplementary Table 1.

### Tissue preparation and immunofluorescence

For immunofluorescence staining, tissues were fixed with 4% paraformaldehyde in PBS by immersion for 1 h at room temperature. Cochleae were decalcified in 120 mM EDTA overnight at 4 °C. After dissection, the samples were washed in PBS and blocked with 10% donkey serum in 10 mM PBS with 1% Triton X-100 for 1 h at room temperature and incubated with primary antibody overnight at 4 °C. The primary antibodies were rabbit anti-MYOSIN VIIa (MYO7A, 1: 800, Proteus Biosciences, Cat# 25-6790), goat anti-SOX2 (1:500, Santa Cruz Biotechnology, Cat# sc-17320), goat anti-Prestin (1:300, Santa Cruz Biotechnology, Cat# sc-22692), rabbit anti-VGLUT3 (1:300, Synaptic Systems, Cat# 135203), mouse anti-PARVALBUMIN (1:800, Sigma, Cat# P3088), mouse anti-TUJ1 (1:1000, Sigma, Cat#T2200), mouse anti-Oncomodulin (OCM, 1:300, Santa Cruz, Cat# sc-7446), goat anti-ANXA4 (1:200, R&D Systems, Cat# AF4146), and goat anti-SPP1 (1:200, R&D Systems, Cat# AF808). The next day, the appropriate secondary Alexa Flour-conjugated antibodies (1:300, Thermo Fisher Scientific) were incubated at room temperature for 1–2 h. HC bundles were labeled with phalloidin (1:800, Thermo Fisher Scientific), and nuclei were labeled with DAPI (1:1000, Thermo Fisher Scientific).

### Image acquisition and cell counts

High-magnification fluorescent images were acquired using a 63 × objective on a Leica SP8 confocal microscope, while whole utricle images were captured using a 20 × objective on the same confocal microscope. Images were analyzed using Fiji software (v1.53, NIH, Bethesda, Maryland, USA). For utricles and saccules, the numbers of HCs were manually counted per 100 × 100 μm^2^ area in both the striolar and extrastriolar region (Fig. 2C). For every crista, cell counts were performed per 100 × 100 μm^2^ area in the central and peripheral regions. We chose HCs with bundles randomly and measured the heights of its tallest stereocilia in each cells in the utricular striola and extrastriola. Each cochlea was divided into the apical, middle, and basal turns, and two regions of 100 μm length in each turn were randomly chosen and the average number of HCs was calculated for each sample. The number of spiral ganglion neurons in Rosenthal’s canal was counted in each section, and data were obtained from five separate sections in each cochlea to obtain a mean value.

### Statistical analyses

Statistical analyses were performed using GraphPad Prism 8.0 software. Data are expressed as the mean ± SEM, and two-tailed, unpaired Student’s *t*-tests, or one- or two-way ANOVA followed by post hoc analysis via Tukey’s multiple comparisons test were used to determine statistical significance. *P* < 0.05 was considered statistically significant. Details of the experiments can be found in the figures and figure legends.

### Supplementary Information

Below is the link to the electronic supplementary material.

Figure S1 Overexpression of *Atoh1* led to the dysfunction of the vestibular system. (A) The experimental protocol. *Atoh1* overexpression was induced from E13.5 in the vestibule, and mice were sacrificed at P35. (B) Tail-hanging test of control and *Gfi1*^Cre^/*Atoh1*-OE mice at P35. Control mice would reach for the horizontal surface when the tail was held (score 0), while most *Gfi1*^Cre^/*Atoh1*-OE mice bent their bodies ventrally (score 1). (C) The tail-hanging test scores at P35 were significantly different between control and *Gfi1*^Cre^/*Atoh1*-OE mice. (D) Swimming test. Control mice could swim normally (score 0), while *Gfi1*^Cre^/*Atoh1*-OE mice would swim irregularly (score 1), float immobile (score 2), or tumble underwater (score 3, not shown in the figure). (E) The swimming test scores at P35 were significantly different between control and *Gfi1*^Cre^/*Atoh1*-OE mice. (F) Gain of HAVOR test. The gain of *Gfi1*^Cre^/*Atoh1*-OE was significantly decreased compared with the control group at all frequencies. (G) Comparison of the amplitude of eye movement in *Gfi1*^Cre^ and *Gfi1*^Cre^/*Atoh1*-OE mice in the OVAR test. Data in C, E, F, and G are presented as the mean ± S.E.M. Wilcoxon test in C and E. Unpaired t-test in G. Two-way ANOVA in F. *p < 0.05, **p < 0.01, *** *p* < 0.001, **** *p* < 0.0001. Supplementary file1 (TIF 4292 KB)

Figure S2 *Atoh1* overexpression did not result in HC loss in the vestibular epithelium. (A) The density of MYO7A^+^ HCs was comparable in the saccule of control and *Gfi1*^Cre^/*Atoh1*-OE mice at P35. (B) The density of MYO7A^+^ HCs was comparable in the crista of control and *Gfi1*^Cre^/*Atoh1*-OE mice at P35. (C) Quantification of HCs per 10,000 μm^2^ in the striola and extrastriola of the utricle at P15 and P35. (D) Quantification of HCs per 10,000 μm^2^ in the striola and extrastriola of the saccule at P15 and P35. (E) Quantification of HCs per 10,000 μm^2^ in the crista's central and peripheral zone at P15 and P35. Scale bars: 50 μm and 25 μm in A, 25 μm in B. Data in C-E are presented as the mean ± S.E.M. Two-way ANOVA. Supplementary file2 (TIF 5680 KB)

Figure S3 *Atoh1* overexpression interfered with type I HC differentiation in vestibular organs. (A) Co-immunolabeling of MYO7A^+^ and OCM in the saccule. OCM^+^ HCs decreased significantly in the striola of the *Gfi1*^Cre^/*Atoh1*-OE mouse saccule. (B) Co-immunolabeling of MYO7A^+^ and OCM in the crista. OCM^+^ HCs decreased significantly in the central zone of *Gfi1*^Cre^/*Atoh1*-OE mouse cristae. (C) Comparison of OCM^+^/ MYO7A^+^ HCs per 10,000 μm^2^ in the striola of the saccule and the central zone of the cristae. (D) Co-immunolabeling of MYO7A and SPP1 in the utricle. (E) Co-immunolabeling of MYO7A and SPP1 in the saccule. (F) Co-immunolabeling of MYO7A and SPP1 in the cristae. (G) Quantification and comparison of SPP1^+^/ MYO7A^+^ HCs in the extrastriola of the saccules and the cristae's peripheral zone. Scale bars: 50 μm in B, 100 μm in A, B, and D-G. Data in C and H are presented as the mean ± S.E.M. Two-way ANOVA. ****p < 0.0001. Supplementary file3 (TIF 7482 KB)

Figure S4 *Atoh1* overexpression caused type I HCs to gain type II markers. (A) Co-immunolabeling of MYO7A^+^ and SOX2 in the utricle. (B) Co-immunolabeling of MYO7A and SOX2 in the saccule. (C) Co-immunolabeling of MYO7A and SOX2 in cristae. (D) Comparison of the percentage of SOX2^+^/MYO7A^+^ HCs in the saccule and cristae at P15. (E) Co-immunolabeling of MYO7A and ANXA4 in the utricle. (F) Co-immunolabeling of MYO7A and ANXA4 in the saccule. (G) Co-immunolabeling of MYO7A and ANXA4 in the cristae. (H) Quantification and comparison of the percentage of ANXA4^+^/ MYO7A^+^ HCs in the saccule and cristae. Scale bars: 50 μm in B and C, and 100 μm in others. Data in D and H are presented as the mean ± S.E.M. Two-way ANOVA. ****p < 0.0001. Supplementary file4 (TIF 10996 KB)

Figure S5 Overexpression of *Atoh1* did not affect the fate determination of HCs during the embryonic stage in the cochlea. (A) The experimental protocol. *Atoh1* overexpression was induced from E15.5, and mice were sacrificed at P2 and P7. (B) MYO7A^+^ HCs of the cochlea at P2. No HC loss was seen in *Gfi1*^Cre^/*Atoh1*-OE mice. (C) Hair bundles of HCs by staining for phalloidin at P2. (D) Hair bundles of HCs by staining for phalloidin at P7. (E) Staining of MYO7A and SOX2 at P7. No SOX2^+^/MYO7A^+^ HCs were observed. (F) Quantification of MYO7A^+^ IHCs and OHCs in the cochleae at P2. (G) Quantification of MYO7A^+^ IHCs and OHCs in the cochleae at P7. Scale bars: 20 μm. Data are presented as the mean ± S.E.M. Two-way ANOVA. Supplementary file5 (TIF 6822 KB)

Figure S6 Overexpression of *Atoh1* disturbed the maturation of HCs during the postnatal period in the cochlea. (A) *Atoh1*CreER mice were crossed with CAG-loxP-stop-loxP-*Atoh1*-HA mice to generate *Atoh1*CreER/*Atoh1*-OE mice. (B) The experimental protocol of C-E. *Atoh1* overexpression was induced from P2, and mice were sacrificed at P7. (C) Comparison of IHCs and OHCs of *Atoh1*CreER/*Atoh1*-OE mice at P7. (D) Co-immunolabeling of Prestin and VGLUT3 with PARVALBUMIN at P7. The staining of Prestin and VGLUT3 was weak in *Atoh1*CreER/*Atoh1*-OE mice. (E) Relative mRNA expression at P7. (F) *Prestin*^CreER^ mice were crossed with CAG-loxP-stop-loxP-*Atoh1*-HA mice to generate PrestinCreER/*Atoh1*-OE mice. (G) The experimental protocol of H-I. *Atoh1* overexpression was induced from P7, and mice were sacrificed at P25. (H) Co-immunolabeling of PARVALBUMIN and Prestin. Scattered HC loss and uneven staining of Prestin were observed in the cochlea of *Prestin*^CreER^/*Atoh1*-OE mice at P25. (I) OHC diameter and length of control and *Prestin*^CreER^/*Atoh1*-OE mice at P25. Scale bars: 20 μm. Data in C, E, and I are presented as the mean ± S.E.M. Two-way ANOVA in C. Unpaired t-test in E and I. ***p < 0.001. ****p < 0.0001. Supplementary file6 (TIF 4933 KB)

Figure S7 *Atoh1* overexpression led to progressive HC death and hearing loss. (A) The experimental protocol of B-C. *Atoh1* overexpression was induced from P2, and mice were sacrificed at P11. (B) HCs in the cochlea of control and *Atoh1*^CreER^/*Atoh1*-OE mice at P11. (C) Comparison of IHCs and OHCs at P11. (D) The experimental protocol of E and F. *Atoh1* overexpression was induced from P7, and mice were sacrificed at P21, P25, P35, and P53. (E) ABR thresholds of control and *Prestin*^CreER^/*Atoh1*-OE mice at P25 and P35. (F) MYO7A staining showed no HC loss in the cochleae of control mice. Scattered HC loss was seen in *Prestin*^CreER^/*Atoh1*-OE cochleae at P21. Massive HC loss was seen in *Prestin*^CreER^/*Atoh1*-OE mouse cochleae at P35. Few OHCs were observed in *Prestin*^CreER^/*Atoh1*-OE mouse cochleae at P53. (G) Quantification of the HC number from the apex to the base at P21, P25, P35, and P53. Scale bars: 20 μm. Data in C and G are presented as the mean ± S.E.M. Two-way ANOVA. *p < 0.05, **p < 0.01, *** *p*<0.01， ****p < 0.0001. Supplementary file7 (TIF 4390 KB)

Figure S8 *Atoh1* overexpression did not interfere with the nerve fiber and spiral ganglia neurons. (A-B) The spiral ganglion neurons and nerve fibers of control and *Gfi1*^Cre^/*Atoh1*-OE mice in the cochlea at P35. (C) Comparison of the number of SGNs of Rosenthal’s canal in the cochlea at P35. (D) The nerve fibers of vestibular organs. Scale bars: 50 μm. Data in C are presented as the mean ± S.E.M. Unpaired *t*-test. Supplementary file 8 (TIF 5357 KB)

Supplementary file 9 (DOCX 14 KB)

## Data Availability

The datasets generated and analyzed in the current study are available from the corresponding author on reasonable request. The manuscript has data included as electronic supplementary material.
