# Supplementary figures and images for: The proper timing of Atoh1 expression is pivotal for hair cell subtype differentiation and the establishment of inner ear function
Source: Cell Mol Life Sci. 2023 Nov 6;80(12):349. doi: 10.1007/s00018-023-04947-w (PMC10628023; doi:10.1007/s00018-023-04947-w)

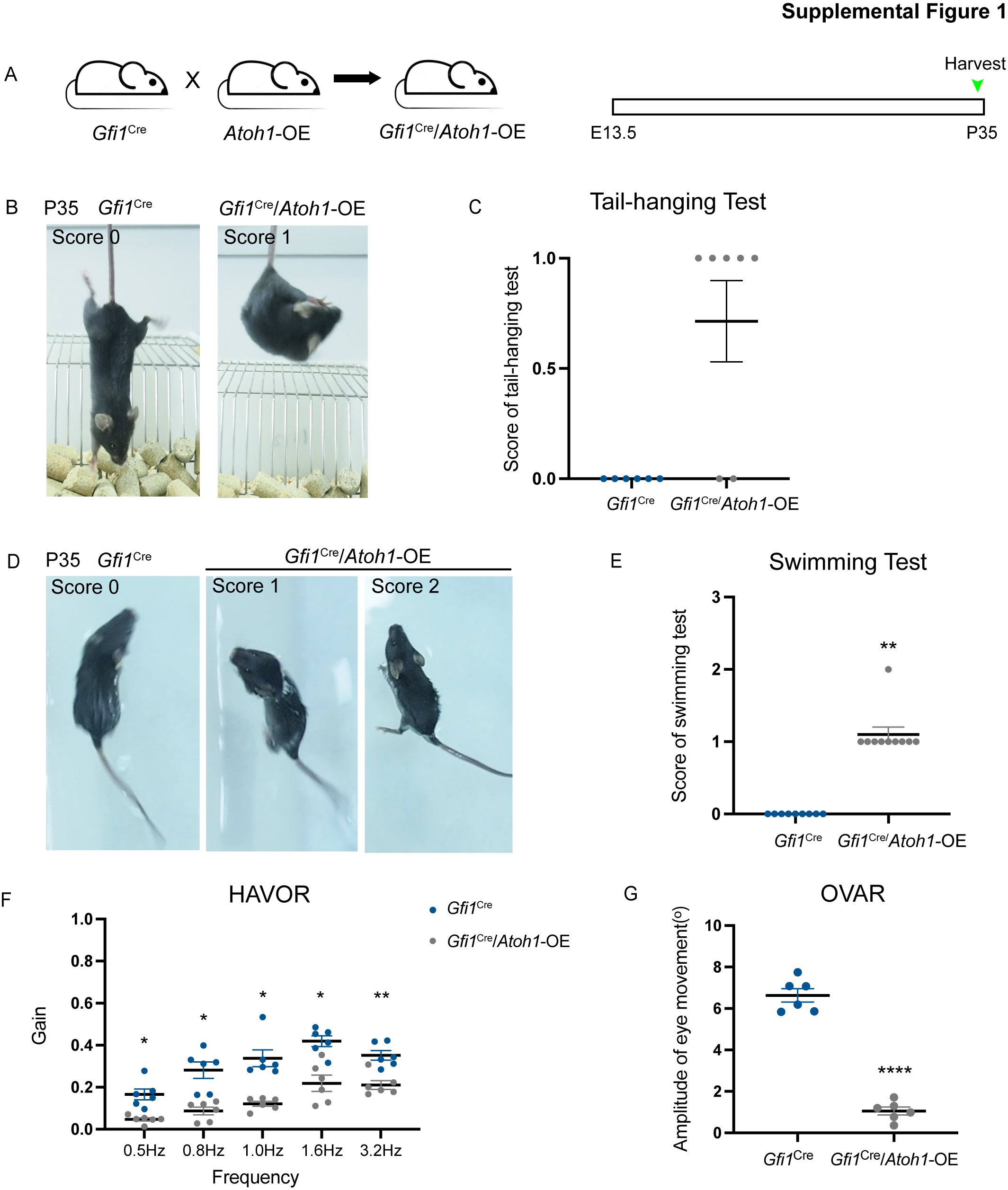

Supplement: Supplementary file 1 — Overexpression of Atoh1 led to the dysfunction of the vestibular system. (A) The experimental protocol. Atoh1 overexpression was induced from E13.5 in the vestibule, and mice were sacrificed at P35. (B) Tail-hanging test of control and Gfi1Cre/Atoh1-OE mice at P35. Control mice would reach for the horizontal surface when the tail was held (score 0), while most Gfi1Cre/Atoh1-OE mice bent their bodies ventrally (score 1). (C) The tail-hanging test scores at P35 were significantly different between control and Gfi1Cre/Atoh1-OE mice. (D) Swimming test. Control mice could swim normally (score 0), while Gfi1Cre/Atoh1-OE mice would swim irregularly (score 1), float immobile (score 2), or tumble underwater (score 3, not shown in the figure). (E) The swimming test scores at P35 were significantly different between control and Gfi1Cre/Atoh1-OE mice. (F) Gain of HAVOR test. The gain of Gfi1Cre/Atoh1-OE was significantly decreased compared with the control group at all frequencies. (G) Comparison of the amplitude of eye movement in Gfi1Cre and Gfi1Cre/Atoh1-OE mice in the OVAR test. Data in C, E, F, and G are presented as the mean ± S.E.M. Wilcoxon test in C and E. Unpaired t-test in G. Two-way ANOVA in F. *p < 0.05, **p < 0.01, *** p < 0.001, **** p < 0.0001. Supplementary file1 (TIF 4292 KB) [file 18_2023_4947_MOESM1_ESM.tif]

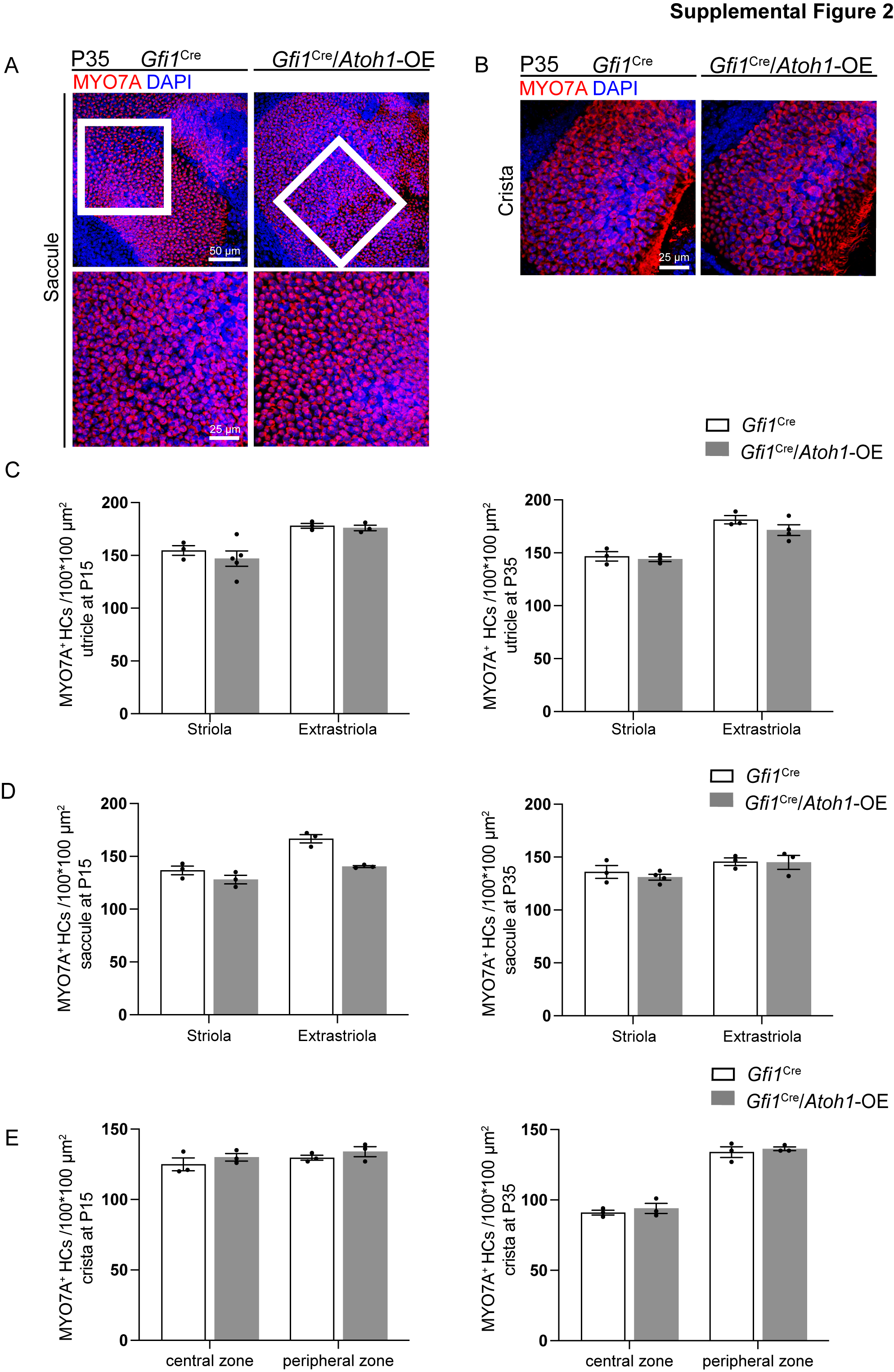

Supplement: Supplementary file 2 — Atoh1 overexpression did not result in HC loss in the vestibular epithelium. (A) The density of MYO7A+ HCs was comparable in the saccule of control and Gfi1Cre/Atoh1-OE mice at P35. (B) The density of MYO7A+ HCs was comparable in the crista of control and Gfi1Cre/Atoh1-OE mice at P35. (C) Quantification of HCs per 10,000 μm2 in the striola and extrastriola of the utricle at P15 and P35. (D) Quantification of HCs per 10,000 μm2 in the striola and extrastriola of the saccule at P15 and P35. (E) Quantification of HCs per 10,000 μm2 in the crista's central and peripheral zone at P15 and P35. Scale bars: 50 μm and 25 μm in A, 25 μm in B. Data in C-E are presented as the mean ± S.E.M. Two-way ANOVA. Supplementary file2 (TIF 5680 KB) [file 18_2023_4947_MOESM2_ESM.tif]

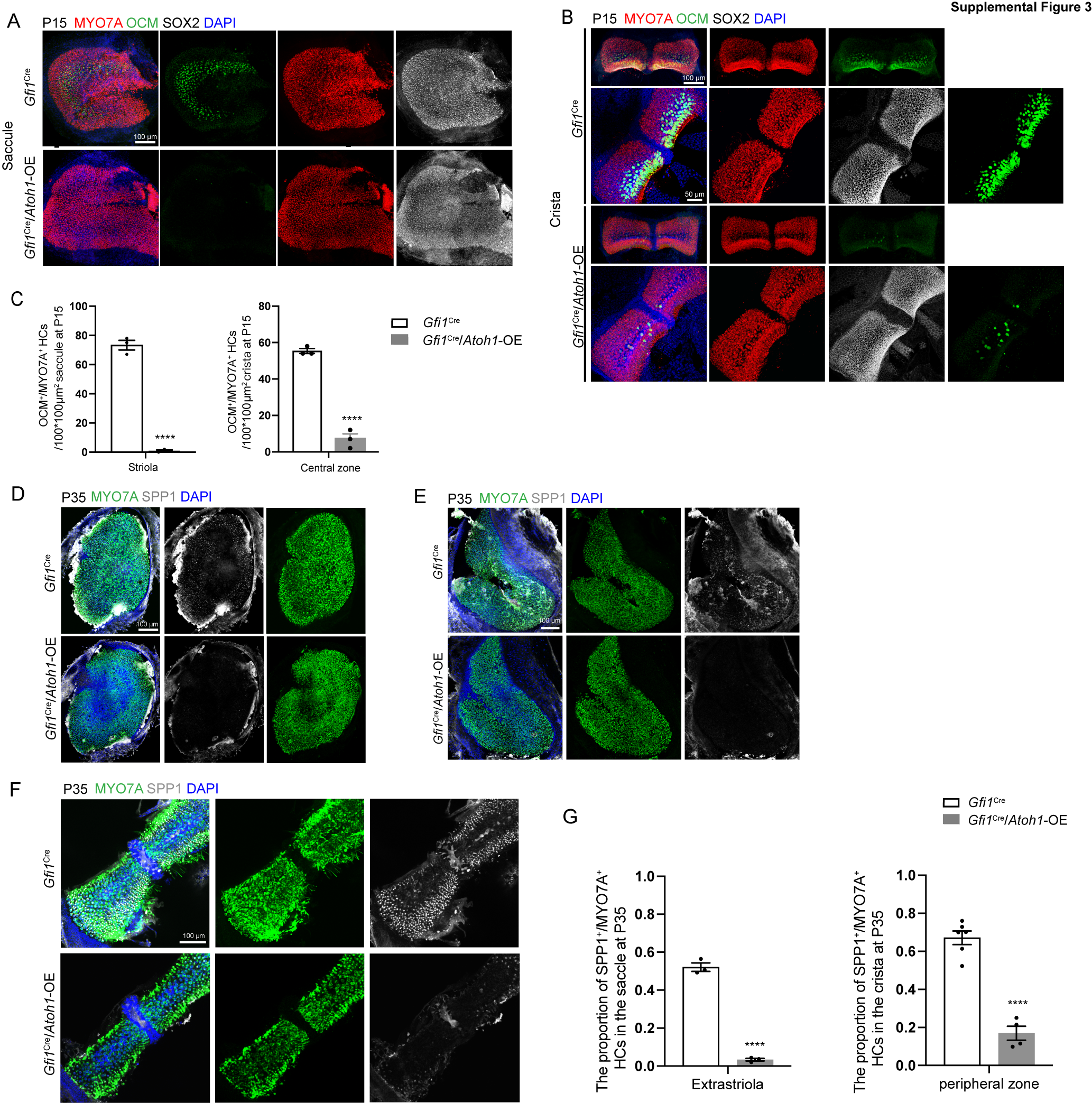

Supplement: Supplementary file 3 — Atoh1 overexpression interfered with type I HC differentiation in vestibular organs. (A) Co-immunolabeling of MYO7A+ and OCM in the saccule. OCM+ HCs decreased significantly in the striola of the Gfi1Cre/Atoh1-OE mouse saccule. (B) Co-immunolabeling of MYO7A+ and OCM in the crista. OCM+ HCs decreased significantly in the central zone of Gfi1Cre/Atoh1-OE mouse cristae. (C) Comparison of OCM+/ MYO7A+ HCs per 10,000 μm2 in the striola of the saccule and the central zone of the cristae. (D) Co-immunolabeling of MYO7A and SPP1 in the utricle. (E) Co-immunolabeling of MYO7A and SPP1 in the saccule. (F) Co-immunolabeling of MYO7A and SPP1 in the cristae. (G) Quantification and comparison of SPP1+/ MYO7A+ HCs in the extrastriola of the saccules and the cristae's peripheral zone. Scale bars: 50 μm in B, 100 μm in A, B, and D-G. Data in C and H are presented as the mean ± S.E.M. Two-way ANOVA. ****p < 0.0001. Supplementary file3 (TIF 7482 KB) [file 18_2023_4947_MOESM3_ESM.tif]

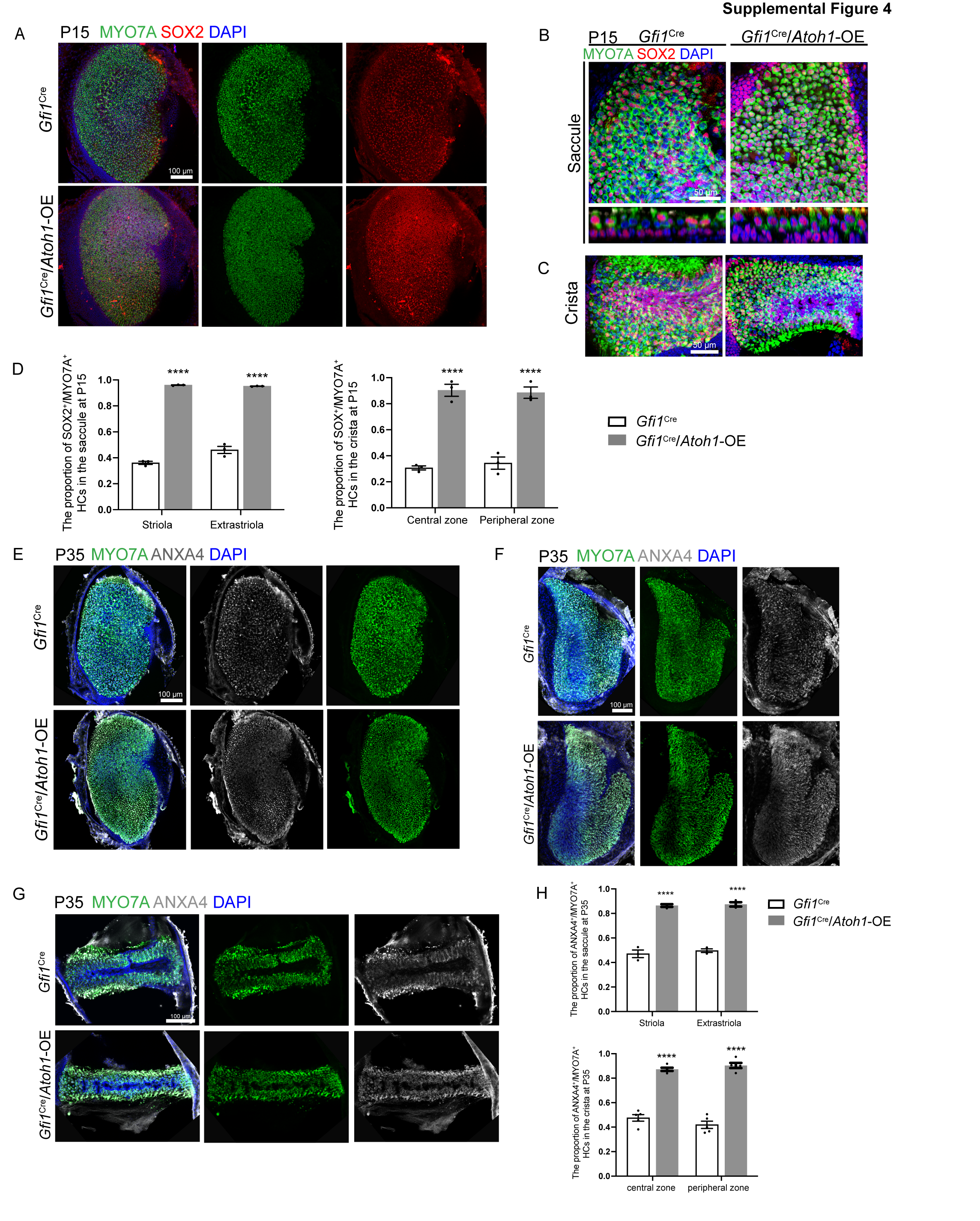

Supplement: Supplementary file 4 — Atoh1 overexpression caused type I HCs to gain type II markers. (A) Co-immunolabeling of MYO7A+ and SOX2 in the utricle. (B) Co-immunolabeling of MYO7A and SOX2 in the saccule. (C) Co-immunolabeling of MYO7A and SOX2 in cristae. (D) Comparison of the percentage of SOX2+/MYO7A+ HCs in the saccule and cristae at P15. (E) Co-immunolabeling of MYO7A and ANXA4 in the utricle. (F) Co-immunolabeling of MYO7A and ANXA4 in the saccule. (G) Co-immunolabeling of MYO7A and ANXA4 in the cristae. (H) Quantification and comparison of the percentage of ANXA4+/ MYO7A+ HCs in the saccule and cristae. Scale bars: 50 μm in B and C, and 100 μm in others. Data in D and H are presented as the mean ± S.E.M. Two-way ANOVA. ****p < 0.0001. Supplementary file4 (TIF 10996 KB) [file 18_2023_4947_MOESM4_ESM.tif]

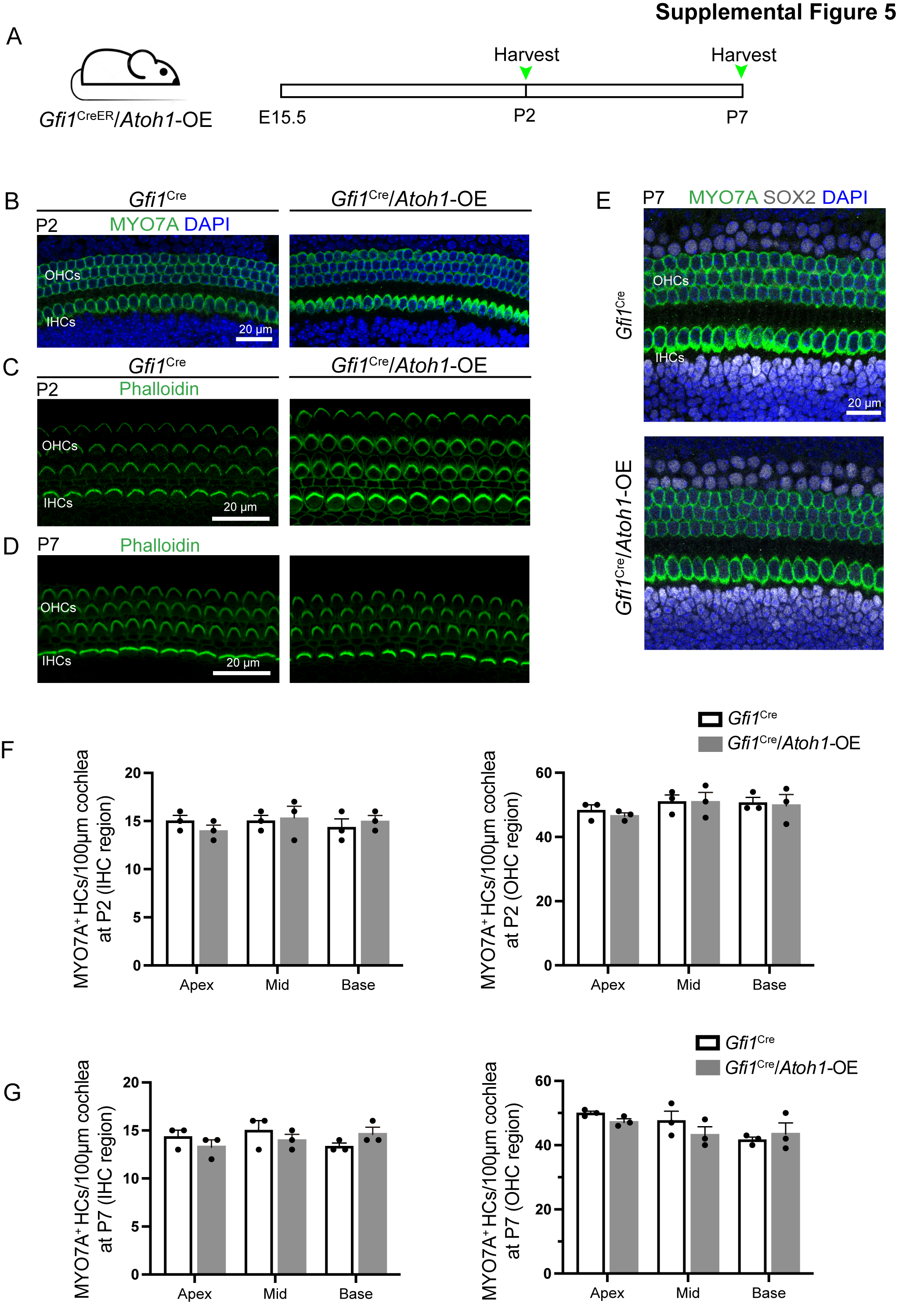

Supplement: Supplementary file 5 — Overexpression of Atoh1 did not affect the fate determination of HCs during the embryonic stage in the cochlea. (A) The experimental protocol. Atoh1 overexpression was induced from E15.5, and mice were sacrificed at P2 and P7. (B) MYO7A+ HCs of the cochlea at P2. No HC loss was seen in Gfi1Cre/Atoh1-OE mice. (C) Hair bundles of HCs by staining for phalloidin at P2. (D) Hair bundles of HCs by staining for phalloidin at P7. (E) Staining of MYO7A and SOX2 at P7. No SOX2+/MYO7A+ HCs were observed. (F) Quantification of MYO7A+ IHCs and OHCs in the cochleae at P2. (G) Quantification of MYO7A+ IHCs and OHCs in the cochleae at P7. Scale bars: 20 μm. Data are presented as the mean ± S.E.M. Two-way ANOVA. Supplementary file5 (TIF 6822 KB) [file 18_2023_4947_MOESM5_ESM.tif]

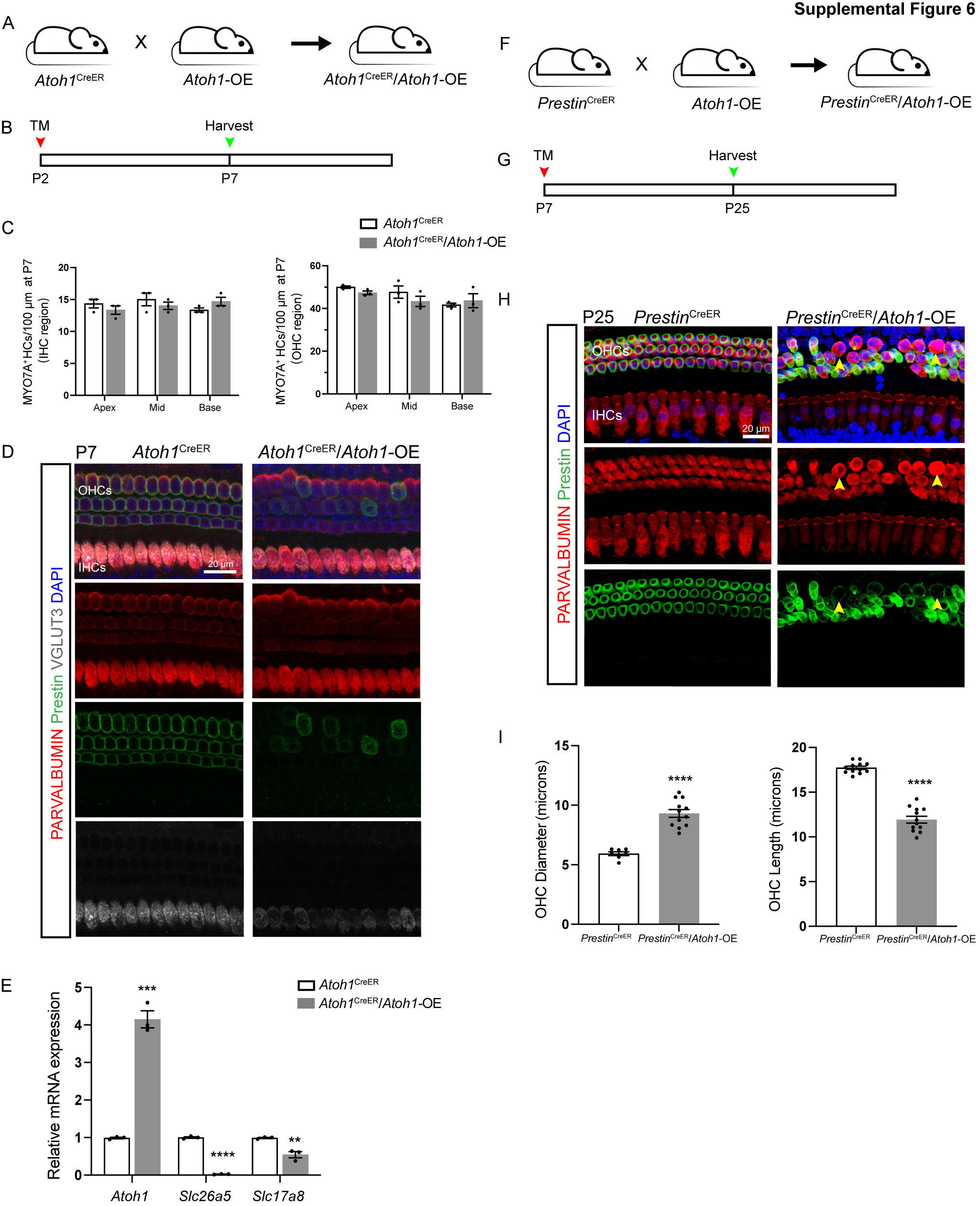

Supplement: Supplementary file 6 — Overexpression of Atoh1 disturbed the maturation of HCs during the postnatal period in the cochlea. (A) Atoh1CreER mice were crossed with CAG-loxP-stop-loxP-Atoh1-HA mice to generate Atoh1CreER/Atoh1-OE mice. (B) The experimental protocol of C-E. Atoh1 overexpression was induced from P2, and mice were sacrificed at P7. (C) Comparison of IHCs and OHCs of Atoh1CreER/Atoh1-OE mice at P7. (D) Co-immunolabeling of Prestin and VGLUT3 with PARVALBUMIN at P7. The staining of Prestin and VGLUT3 was weak in Atoh1CreER/Atoh1-OE mice. (E) Relative mRNA expression at P7. (F) PrestinCreER mice were crossed with CAG-loxP-stop-loxP-Atoh1-HA mice to generate PrestinCreER/Atoh1-OE mice. (G) The experimental protocol of H-I. Atoh1 overexpression was induced from P7, and mice were sacrificed at P25. (H) Co-immunolabeling of PARVALBUMIN and Prestin. Scattered HC loss and uneven staining of Prestin were observed in the cochlea of PrestinCreER/Atoh1-OE mice at P25. (I) OHC diameter and length of control and PrestinCreER/Atoh1-OE mice at P25. Scale bars: 20 μm. Data in C, E, and I are presented as the mean ± S.E.M. Two-way ANOVA in C. Unpaired t-test in E and I. ***p < 0.001. ****p < 0.0001. Supplementary file6 (TIF 4933 KB) [file 18_2023_4947_MOESM6_ESM.tif]

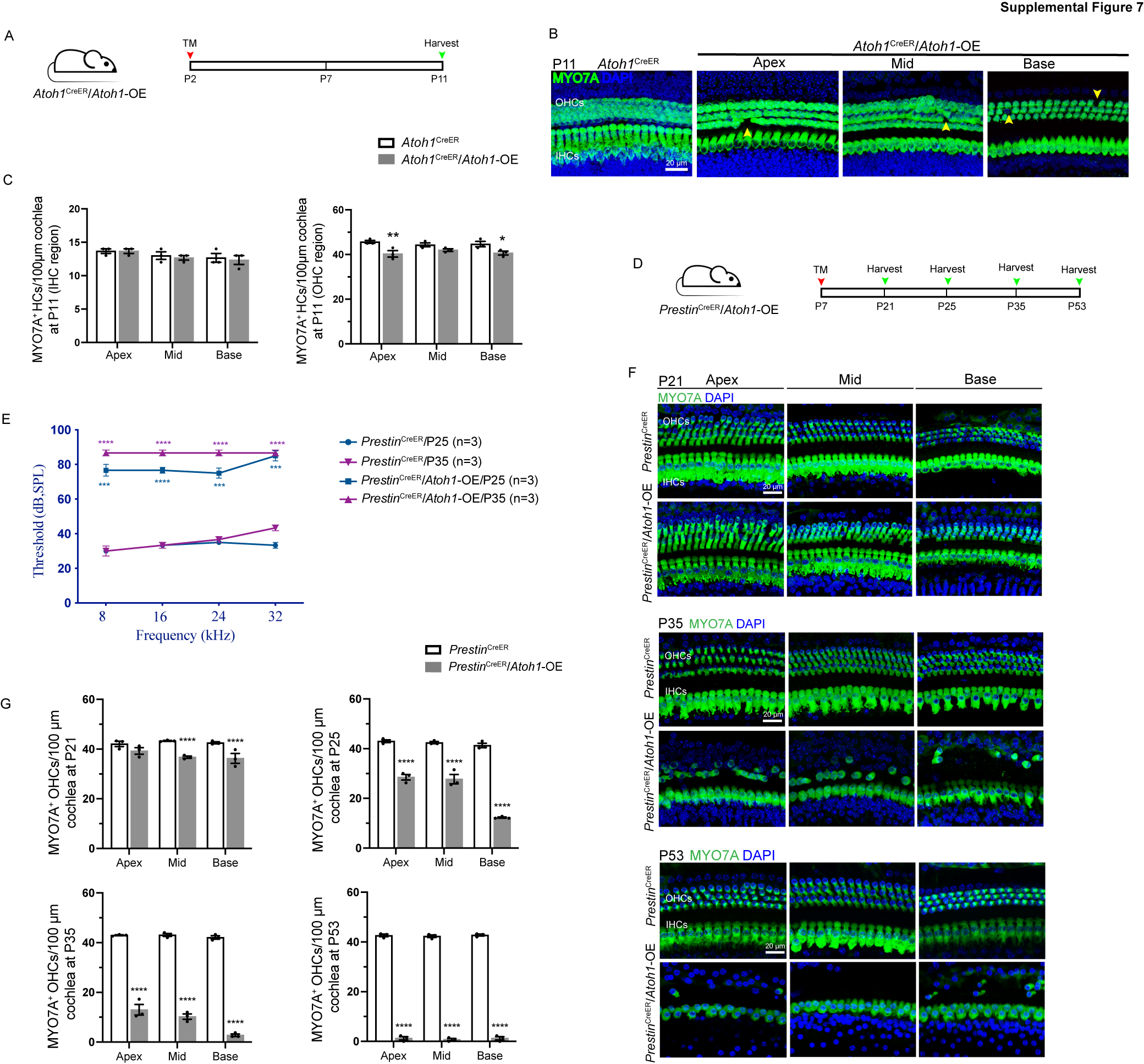

Supplement: Supplementary file 7 — Atoh1 overexpression led to progressive HC death and hearing loss. (A) The experimental protocol of B-C. Atoh1 overexpression was induced from P2, and mice were sacrificed at P11. (B) HCs in the cochlea of control and Atoh1CreER/Atoh1-OE mice at P11. (C) Comparison of IHCs and OHCs at P11. (D) The experimental protocol of E and F. Atoh1 overexpression was induced from P7, and mice were sacrificed at P21, P25, P35, and P53. (E) ABR thresholds of control and PrestinCreER/Atoh1-OE mice at P25 and P35. (F) MYO7A staining showed no HC loss in the cochleae of control mice. Scattered HC loss was seen in PrestinCreER/Atoh1-OE cochleae at P21. Massive HC loss was seen in PrestinCreER/Atoh1-OE mouse cochleae at P35. Few OHCs were observed in PrestinCreER/Atoh1-OE mouse cochleae at P53. (G) Quantification of the HC number from the apex to the base at P21, P25, P35, and P53. Scale bars: 20 μm. Data in C and G are presented as the mean ± S.E.M. Two-way ANOVA. *p < 0.05, **p < 0.01, *** p<0.01， ****p < 0.0001. Supplementary file7 (TIF 4390 KB) [file 18_2023_4947_MOESM7_ESM.tif]

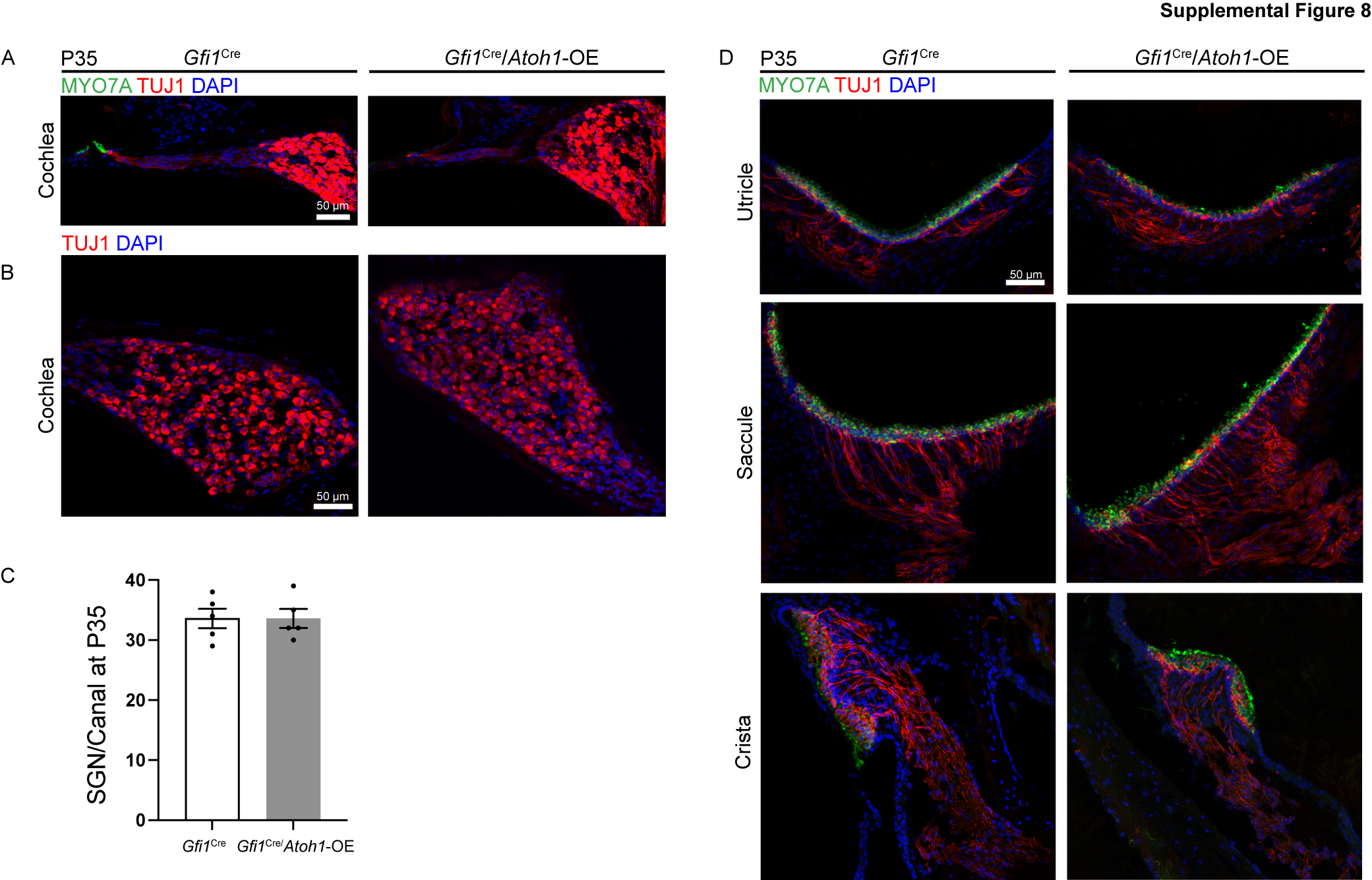

Supplement: Supplementary file 8 — Atoh1 overexpression did not interfere with the nerve fiber and spiral ganglia neurons. (A-B) The spiral ganglion neurons and nerve fibers of control and Gfi1Cre/Atoh1-OE mice in the cochlea at P35. (C) Comparison of the number of SGNs of Rosenthal’s canal in the cochlea at P35. (D) The nerve fibers of vestibular organs. Scale bars: 50 μm. Data in C are presented as the mean ± S.E.M. Unpaired t-test. Supplementary file 8 (TIF 5357 KB) [file 18_2023_4947_MOESM8_ESM.tif]
