# Supplementary material for: The proper timing of Atoh1 expression is pivotal for hair cell subtype differentiation and the establishment of inner ear function
Source: Cell Mol Life Sci. 2023 Nov 6;80(12):349. doi: 10.1007/s00018-023-04947-w (PMC10628023; doi:10.1007/s00018-023-04947-w)
Supplement: Supplementary file 9 — (DOCX 14 KB) [file 18_2023_4947_MOESM9_ESM.docx]

**Supplemental table**

Supplemental table 1 The primers used in qPCR.

|  |  |  |
| --- | --- | --- |
| Atoh1 | 5’-TGGGGTACAGAAGCAAAGGA-3’ | 5’-TCCGACAGAGCGTTGATGTA-3’ |
| Slc26a5 | 5’-AAGCAGGCATTCACGTGTAC-3’ | 5’-CTTGGGGAAGCTGGAGTACC-3’ |
| Slc17a8 | 5’-GGATCTCAAATGGTGTGGGG-3’ | 5’-GCGAAGACCCCGTAGAAGAT-3’ |
| Sox2 | 5’-CTCCGGGACATGATCAGCA-3’ | 5’-CTCTCCCCTTCTCCAGTTCG-3’ |
| Espn | 5’-GCCCTGCCTATCCACTACG-3’ | 5’-CGTTGTTGGTTTGGGCATTC-3’ |
| Casp3 | 5’-TGACTCTCCTTCCAGATCCCA-3’ | 5’-TGCCCACACTAGGCTGACA-3’ |
| Casp9 | 5’-CCTACTCGAAGACTTACCCAGT-3’ | 5’-GCATTGGGGTGAATGATAGCA-3’ |
| Bax | 5’-CGTGGTTGCCCTCTTCTACT-3’ | 5’-TTGGATCCAGACAAGCAGCC-3’ |
| Bcl2 | 5’-ATCCAGGATAACGGAGGCTG-3’ | 5’-CTTCAGAGACAGCCAGGAGA-3’ |
| Bad | 5’-ACCAGCAGCCCAGAGTATG-3’ | 5’-GGCCCCTATCTGTAGCACTAG-3’ |
| Gapdh | 5’-TGCGACTTCAACAGCAACTC-3’ | 5’-CTTGCTCAGTGTCCTTGCTG-3’ |
